# Indirect Skin Homeostasis Modulation by an Essential Oil Blend in Mice with Insomnia Comorbid Anxiety: Sensitivity of Dermal Fibroblasts to Exogenously Applied Circulating Molecules

**DOI:** 10.3390/ph19101576

**Published:** 2026-10-04

**Authors:** Fei Liu, Jiamin Xu, Zimeng Chen, Lei Yao, Yuhong Li, Tiange Wang, Qian Wang, Jing He, Suzhen Yang, Wencui Wang

**Affiliations:** 1 Shandong Freda Biotech Co., Ltd., No. 888 Xinluo Street, High-Tech Zone, Jinan 250101, China; 2 Department of Landscape Architecture, School of Design, Shanghai Jiao Tong University, No. 800 Dongchuan Road, Shanghai 200240, Chinagracelily@sjtu.edu.cn (Y.L.); 3 Xinjiang Eprhan Spice Co., Ltd., No. 567 South Tianshan Road, Kekedala 835219, China

**Keywords:** essential oil blend, insomnia comorbid anxiety, emotional skincare, oxidative stress, extracellular matrix

## Abstract

**Background/Objectives:** Studies have confirmed that essential oils extracted from aromatic plants exert mood-regulating and skin-beneficial effects. Although the concept of “emotional skincare” has attracted growing research interest, experimental evidence supporting the indirect skin-protective effects of essential oils through emotional regulation remains limited. This study investigates the indirect effect of an essential oil blend (EOB) on skin homeostasis in mice with insomnia comorbid anxiety (ICA, a pharmacologically validated emotional stress model induced by para-chlorophenylalanine) and aims to profile the sensitivity of dermal fibroblasts to candidate circulating molecules identified from EOB-treated ICA mice, thereby laying a mechanistic foundation for emotional skincare research. **Methods:** An essential oil blend (EOB) comprising lavender, Kushui rose, sweet orange, Roman chamomile, sandalwood, and clary sage essential oils was prepared. An ICA mouse model was established using para-chlorophenylalanine. Behavioral phenotypes, as well as neurotransmitter and hormonal indices, were measured to validate the anxiolytic and sleep disturbance-alleviating effects of EOB administered via intraperitoneal injection at 300 mg/kg per injection (two injections overall). Skin histological alterations and circulating biomarker profiles were evaluated, and selected differentially altered circulating molecules were exogenously applied to cultured dermal fibroblasts in vitro to assess their effects on fibroblast functions. **Results:** EOB significantly alleviated ICA-like behaviors and ameliorated abnormal levels of brain neurotransmitters and hormones in mice. Although only mild histological variations were observed in the skin after EOB intervention, significant alterations in cortisol, melatonin, 5-hydroxytryptamine, interleukin-10, and type I collagen were detected in serum or skin tissues. Exogenous application of these circulating molecules elicited differential responses in dermal fibroblasts, affecting oxidative stress, inflammatory responses, and extracellular matrix synthesis in a molecule-specific manner. Notably, these fibroblast assays only demonstrate cellular sensitivity in vitro and do not prove that these circulating molecules directly mediate the in vivo effects observed in mice. **Conclusions:** EOB intervention ameliorates insomnia- and anxiety-related behaviors and concurrent favorable changes in skin-associated molecular profiles in ICA mice. These changes are accompanied by alterations in circulating bioactive molecules to which dermal fibroblasts exhibit measurable sensitivity in vitro. These observations are consistent with, but do not definitively establish, a brain–skin axis-mediated mechanism. These findings provide preliminary experimental evidence for the emotional skincare concept and identify candidate circulating molecules for future causal validation; the in vivo regulatory roles of these molecules and the precise signaling pathways involved remain to be established in dedicated mechanistic studies.

## 1. Introduction

Sleep is an essential determinant of human physical and mental well-being. Chronic sleep deprivation impairs immune homeostasis, triggers emotional disturbances, and increases the risk of metabolic disorders and endocrine dysfunction [1,2,3]. Globally, approximately 10–30% of adults suffer from sleep insufficiency [4], with 16% estimated to have insomnia [5]. A large number of insomnia patients present with comorbid depressive and anxiety symptoms [6]. Insomnia and anxiety form a bidirectional interactive cycle: insomnia aggravates anxiety, while anxiety in turn promotes the onset and recurrence of insomnia. Insomnia comorbid anxiety (ICA) severely impairs patients’ daily life and social functioning, and may even cause adverse mental and physical outcomes [7]. Furthermore, clinical and preclinical studies have demonstrated a close association between negative emotional states (e.g., anxiety and depression) and altered skin health phenotypes [8]. Accumulating psychodermatology evidence highlights the brain–skin axis as a bidirectional neuro-immune-endocrine communication network, suggesting that psychological stress and sleep disturbance may reshape peripheral circulating signaling molecules to potentially alter dermal structure and function [9,10]. Nevertheless, the precise molecular mechanisms linking insomnia-anxiety stress to skin physiological dysregulation remain poorly elucidated, and most reported associations are correlative rather than causally validated in vivo.

Aromatherapy, which utilizes aromatic plants for emotional relaxation and physical conditioning, has a long application tradition [11]. Essential oils are extracted from the leaves, stems, flowers, and roots of aromatic plants, and their volatile components are the major bioactive constituents underlying aromatherapy. Modern pharmacological studies have demonstrated that essential oils exert multiple biological activities, including antimicrobial, antioxidant, anti-inflammatory, analgesic, sedative, anxiolytic and sleep-modulating effects [12,13]. Through terpenoid constituents, essential oils mainly regulate the immune, central nervous and endocrine systems, thereby modulating physical homeostasis and emotional status [14,15,16]. Common administration routes include inhalation, topical application, oral delivery and intraperitoneal injection, chosen according to experimental or therapeutic objectives. Multiple studies have demonstrated that essential oils from lavender (*Lavandula angustifolia* Mill., Report S1), Kushui rose (*Rosa sertata* × *Rosa rugosa*, Report S5), sweet orange (*Citrus sinensis* (L.) Osbeck, Report S2), Roman chamomile (*Chamaemelum nobile* (L.) All., Report S4), sandalwood (*Santalum album* L., Report S6), and clary sage (*Salvia sclarea* L., Report S3) exert anxiolytic and sedative effects in insomnia and mood disorders [17,18,19,20,21,22]. Essential oils exert dual regulatory effects: they alleviate emotional disturbances and improve skin physiology when applied topically. They have been documented to function as anti-aging [23,24], whitening [25,26], anti-inflammatory [27,28], hydrating [29], wound-healing [30], and even sunscreen agents [31]. Collectively, existing evidence indicates that essential oils show promise in both mood regulation and skin care research.

The concept of “emotional skincare” has emerged alongside the development of aromacosmetics. Traditional Chinese medicine has long linked emotional status to skin complexion [32]. However, most current studies on the emotion–skin relationship are limited to observational correlation, with no definitive causal evidence or clear mechanistic insight into how emotional disturbances and sleep disorders alter skin homeostasis via circulating molecular mediators. Although essential oil blends have been reported to exert dual modulatory effects on emotional states and skin conditions, the molecular pathways linking ICA progression, EOB intervention, and skin microenvironmental remodeling remain poorly understood and require further mechanistic investigation.

We hypothesize that a six-plant essential-oil blend (EOB) may indirectly alleviate ICA-associated skin dysfunction by modulating circulating stress-related and neuroendocrine molecules, which could potentially modulate dermal fibroblast behaviors. Candidate molecules were screened using a predefined statistical threshold (*p* < 0.05) based on three inclusion criteria: (1) involvement in neuroendocrine or inflammatory regulation of skin homeostasis; (2) detectability in peripheral serum; and (3) previously reported bioactivity in dermal fibroblasts. The screening procedure integrated PCPA-induced pathological alterations and EOB-responsive molecular variations, yielding core candidate molecules including cortisol, melatonin, 5-hydroxytryptamine, and IL-10. Specifically, we propose three testable hypotheses: (1) intraperitoneal EOB treatment attenuates ICA-induced behavioral, neurochemical, and endocrine abnormalities; (2) these phenotypic improvements correlate with changes in circulating levels of candidate molecules; and (3) in vitro fibroblast treatment with these factors induces distinct, molecule-specific changes in oxidative stress status, inflammatory responses, and extracellular matrix synthesis. Importantly, these in vitro experiments serve only as sensitivity assays to demonstrate fibroblast responsiveness to altered molecular profiles, and do not prove that these circulating molecules mediate the in vivo skin changes triggered by EOB. This testable framework characterizes the potential modulatory effects of EOB-regulated circulating molecules on fibroblast function, providing preliminary correlative evidence for emotion–skin crosstalk without establishing definitive mechanistic causality.

## 2. Results

### 2.1. Chemical Composition Analysis of EOB

**Figure 1:**
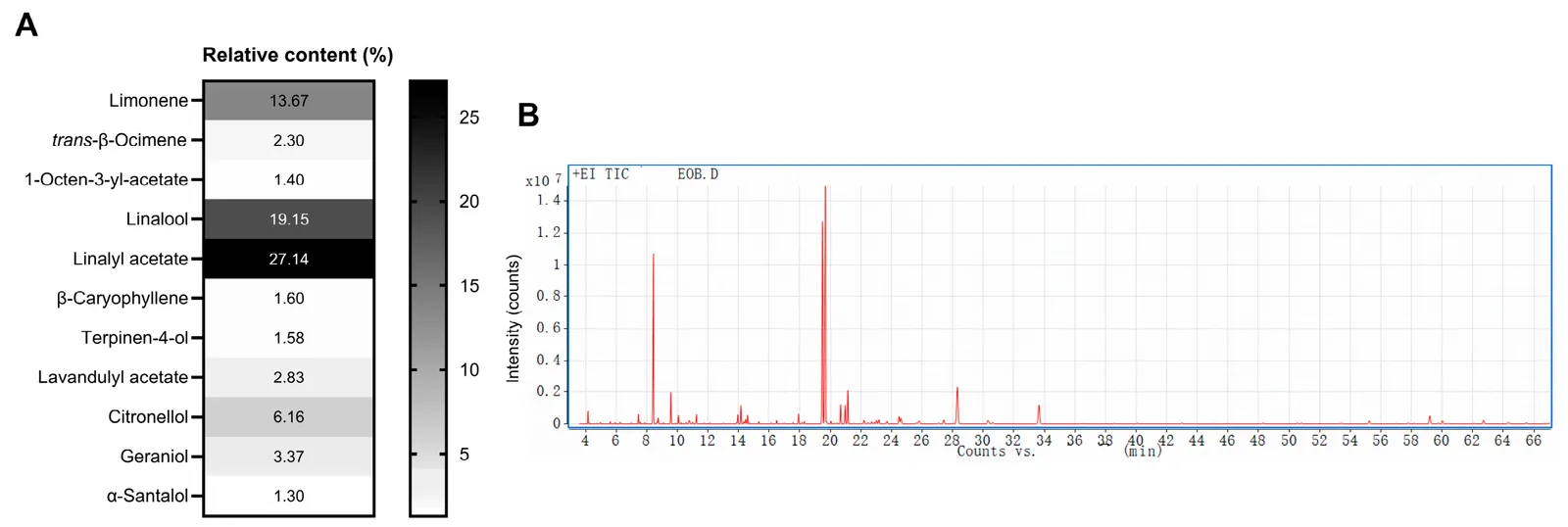
GC-MS-based composition analysis of EOB. (**A**) Heatmap of components with relative content > 1% in EOB. (**B**) Total ion chromatogram of EOB.

In total, 72 volatile components were detected and identified in EOB via GC-MS analysis (Table S1). Of these, 11 components had relative contents above 1% (Figure 1A). The predominant constituents included linalyl acetate (27.14%), linalool (19.15%), limonene (13.64%), and citronellol (6.16%). The total ion chromatogram of EOB is shown in Figure 1B.

### 2.2. Effects of EOB on Sleep in Insomniac Mice

The pentobarbital-induced sleep assay showed that, compared with the CK group, the PCPA group exhibited a significant 31.6% prolongation of sleep latency (*p* = 0.001, *n* = 6), whereas sleep duration showed a non-significant decreasing trend (Table 1). Compared with the PCPA group, the EOB group showed a significant 46.6% reduction in sleep latency (*p* < 0.001, *n* = 6). Meanwhile, EOB treatment markedly increased pentobarbital-induced sleep duration by 721.6% relative to the PCPA group (*p* < 0.01, *n* = 6).

### 2.3. EOB Improves Spontaneous Locomotor Behavior of PCPA-Induced ICA Mice

**Figure 2:**
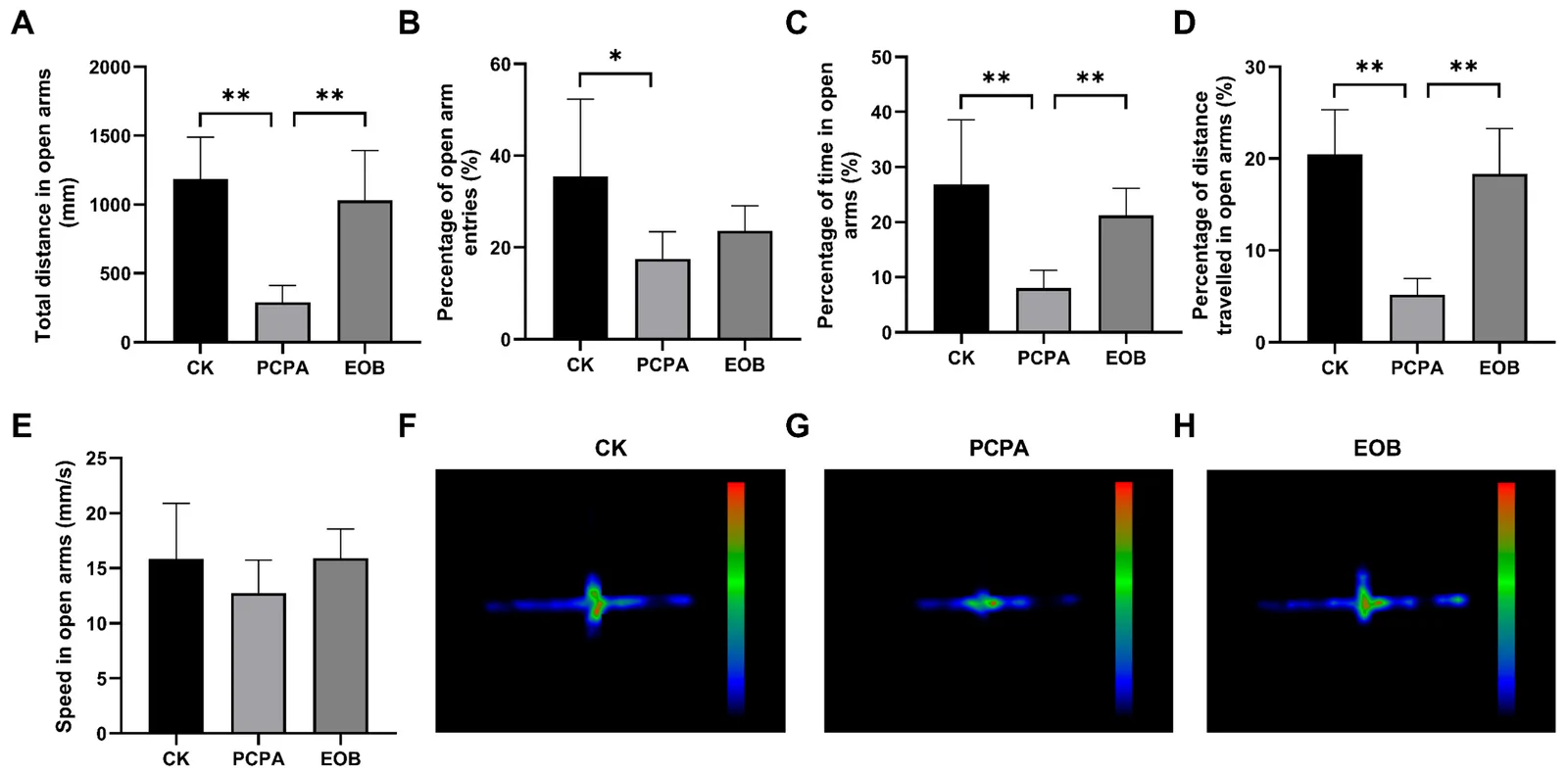
Effects of EOB on spontaneous locomotor-anxiety-related behaviors of PCPA-induced mice in the elevated plus-maze (EPM) test. (**A**) Total distance travelled in open arms, (**B**) percentage of open-arm entries, (**C**) percentage of time spent in open arms, (**D**) percentage of distance travelled in open arms, (**E**) mean speed in open arms. Data are presented as mean ± SD, *n* = 10 independent biological samples per group. Statistical analysis was performed using one-way ANOVA followed by Tukey’s post hoc multiple-comparison test. * *p* < 0.05, ** *p* < 0.01. Horizontal brackets indicate pairwise comparisons vs. the PCPA group. (**F**–**H**) Heatmaps illustrating mice residence frequency across different EPM zones; red denotes high-frequency occupancy, and blue denotes low-frequency occupancy.

In the elevated plus maze (EPM) test, the PCPA group exhibited significant reductions compared with the control group in total distance travelled in open arms (Figure 2A), percentage of open-arm entries (Figure 2B), percentage of time spent in open arms (Figure 2C), and percentage of distance travelled in open arms (Figure 2D), indicating PCPA-induced anxiety-like behavioral alterations. Compared with the PCPA group, EOB intervention markedly increased total distance travelled in open arms, percentage of time spent in open arms, and percentage of distance travelled in open arms. No statistically significant differences were detected between the EOB and PCPA groups regarding the percentage of open-arm entries or open-arm speed (Figure 2E), despite notable directional shifts toward control-group levels in these parameters for the EOB group. These behavioral observations align with the movement trajectories visualized in the residence-frequency heatmaps (Figure 2F–H).

### 2.4. EOB Modulates Brain Neurotransmitter and Hormonal Levels of PCPA-Induced ICA Mice

**Figure 3:**
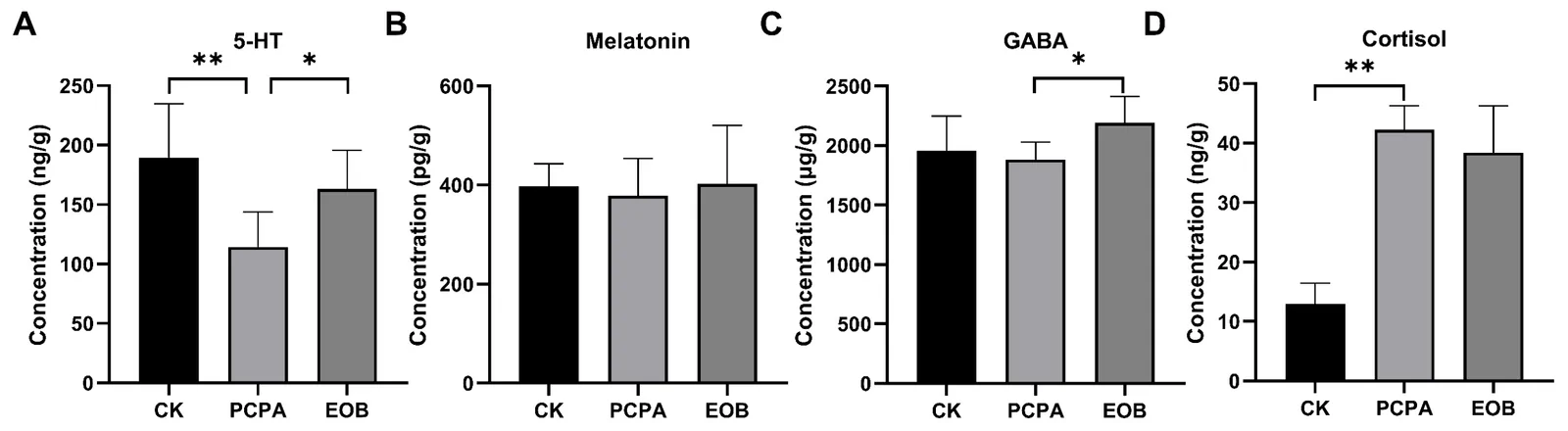
Effects of EOB on neurotransmitter and hormone levels in brain tissues from PCPA-induced mice. (**A**) 5-HT, (**B**) Melatonin, (**C**) GABA, (**D**) Cortisol. Data are presented as mean ± SD, *n* = 10 independent biological samples per group. Statistical analysis was performed using one-way ANOVA followed by Tukey’s post hoc multiple-comparison test. * *p* < 0.05, ** *p* < 0.01. Horizontal brackets indicate pairwise comparisons vs. PCPA group.

Compared with the CK group, PCPA treatment reduced cerebral levels of 5-HT (Figure 3A), melatonin (Figure 3B), and GABA (Figure 3C), although only 5-HT reached statistical significance. By contrast, cerebral cortisol concentration was markedly increased in the PCPA group (Figure 3D). After EOB intervention, cerebral 5-HT and GABA concentrations were significantly increased relative to the PCPA group. Melatonin and cortisol showed trends toward CK-group levels versus the PCPA group, but these alterations did not achieve statistical significance.

### 2.5. EOB Reverses Skin Homeostatic Imbalance in ICA Mice

**Figure 4:**
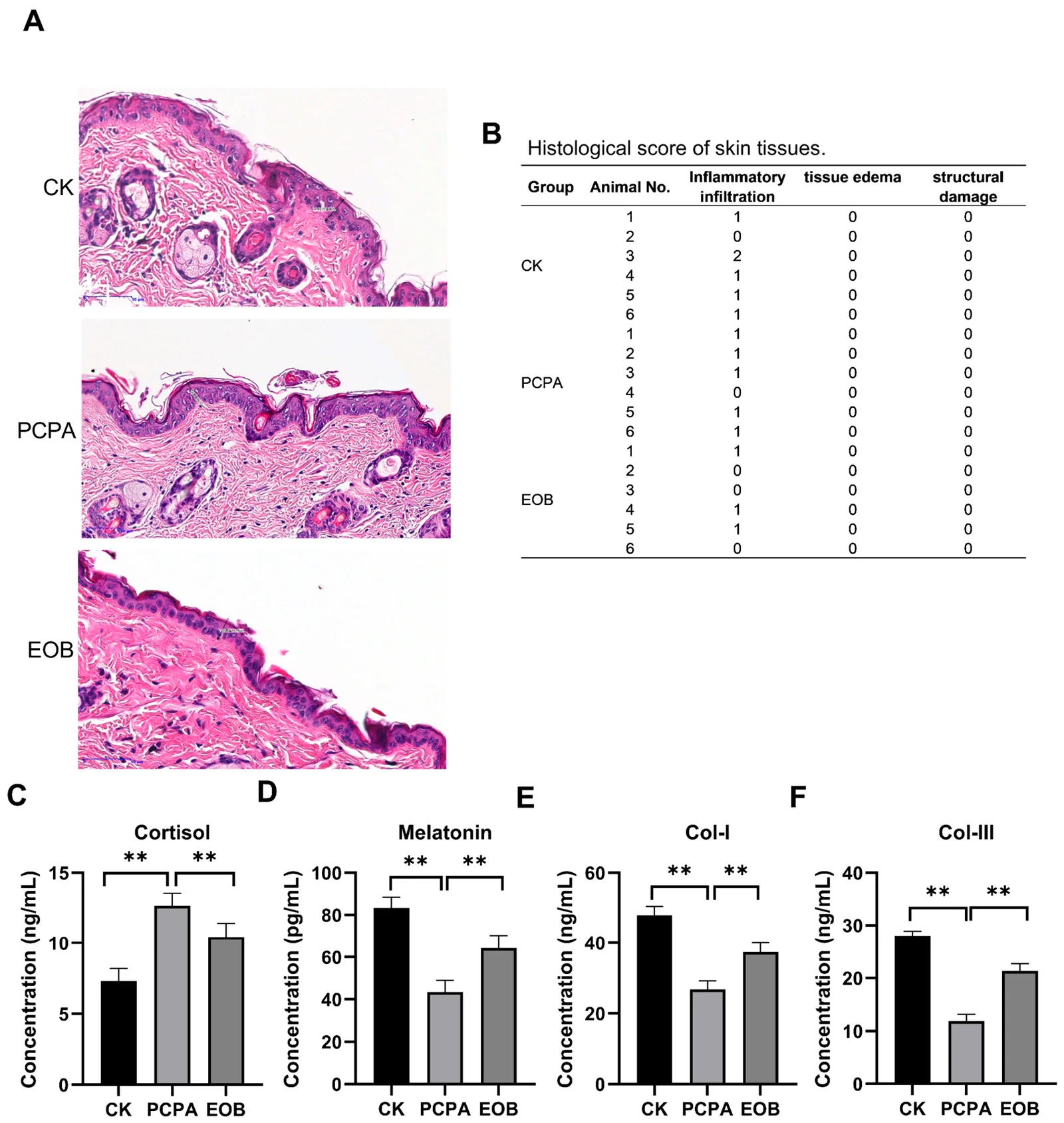
Histopathological changes and skin biochemical alterations in each group. (**A**) Representative H&E-stained micrographs of mouse skin (20× magnification, scale bar = 50 μm). (**B**) Quantitative histopathological scores based on inflammatory infiltration, tissue edema and epidermal structural disruption; five random fields were evaluated per section. Concentrations of (**C**) cortisol, (**D**) melatonin, (**E**) type I collagen (Col-I), and (**F**) type III collagen (Col-III) in skin-tissue homogenates. Data are mean ± SD, *n* = 6 per group. One-way ANOVA with Tukey’s post hoc test. ** *p* < 0.01. Horizontal brackets in panels (**C**–**F**) denote pairwise comparisons vs. PCPA group. Histological scoring was performed independently by two blinded investigators, with individual-sample scores included in quantitative analysis.

H&E staining revealed only sparse inflammatory cell infiltration across all experimental groups (Figure 4A). The histopathological scores were 1.0 ± 0.6 (CK), 0.8 ± 0.4 (PCPA), and 0.5 ± 0.5 (EOB). One-way ANOVA showed no statistically significant inter-group differences (*p* > 0.05). Similarly, the incidence of inflammatory changes was comparable between the CK and PCPA groups (both 83.3%, Figure 4B), indicating that the ICA mouse model did not produce overt histopathological inflammatory lesions in mouse skin. Notably, despite the lack of prominent tissue-level histological alterations, PCPA exposure substantially perturbed skin molecular homeostasis: cutaneous cortisol was significantly upregulated (Figure 4C), whereas melatonin (Figure 4D), Col-I (Figure 4E), and Col-III (Figure 4F) were significantly downregulated. EOB intervention produced significant modulations of these PCPA-driven skin biochemical abnormalities, ameliorating stress-associated skin homeostatic imbalance. Importantly, these beneficial effects were observed exclusively at the molecular-biochemical level; no measurable improvement in skin tissue structure was detected histologically.

### 2.6. EOB Partially Restores Systemic Circulating Molecules in ICA Mice

**Figure 5:**
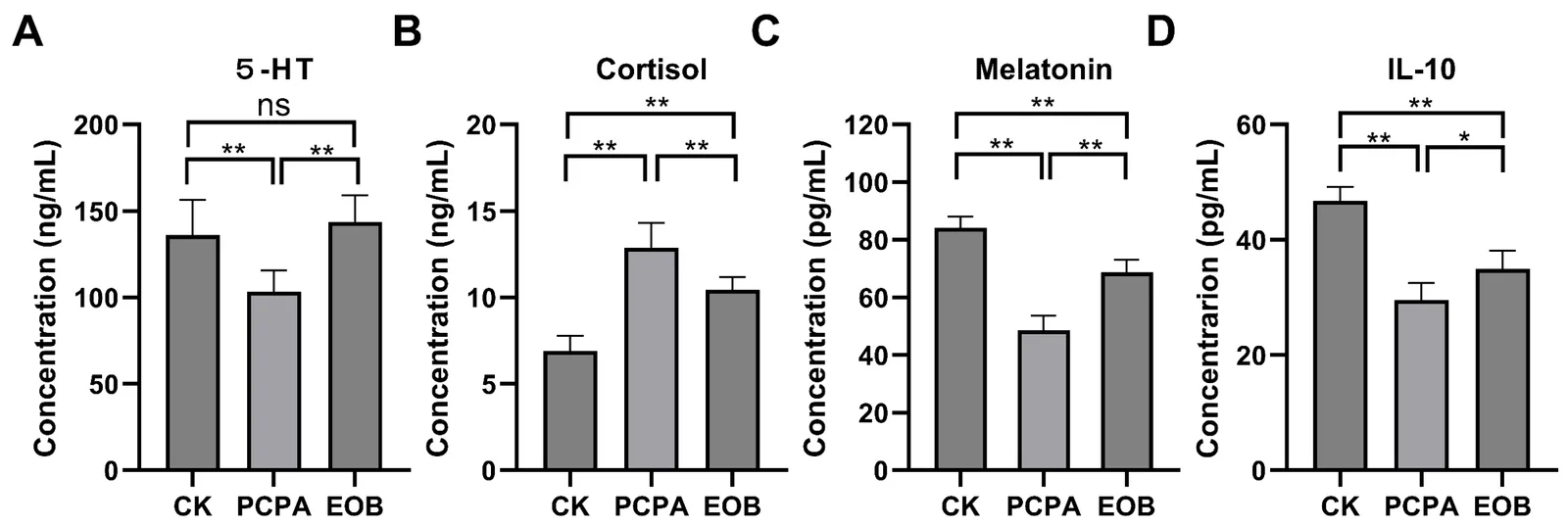
Effects of EOB on serum neurotransmitter, hormone, and inflammatory factor levels in PCPA-treated mice. (**A**) 5-HT, (**B**) Cortisol, (**C**) Melatonin, (**D**) IL-10. Data are mean ± SD. *n* = 10 per group. One-way ANOVA with Tukey’s post hoc test. * *p* < 0.05, ** *p* < 0.01; ns, not significant. Horizontal brackets denote pairwise comparisons vs. CK or PCPA group. All assays were performed using independent biological samples.

Serum levels of neurotransmitters and hormones linked to the sleep–wake cycle and emotional regulation were determined across experimental groups. Compared with the CK group, the PCPA group displayed significantly reduced serum 5-HT and melatonin concentrations, alongside a marked elevation in serum cortisol. Following EOB intervention, serum 5-HT and melatonin were significantly increased, whereas cortisol levels were decreased (Figure 5A–C). These molecular changes indicate systemic neuroendocrine disturbances triggered by PCPA exposure. Together with the behavioral alterations described above, these observations align with the expected phenotypic features of the ICA mouse model and indicate that EOB can modulate circulating molecules associated with sleep–wake and emotional states in this mouse model. Serum IL-10 concentrations were significantly lower in the PCPA group compared with the CK group; EOB intervention produced a significant increase in serum IL-10 (Figure 5D).

### 2.7. EOB Modulates Serum Collagen and MMP Levels in ICA Mice

**Figure 6:**
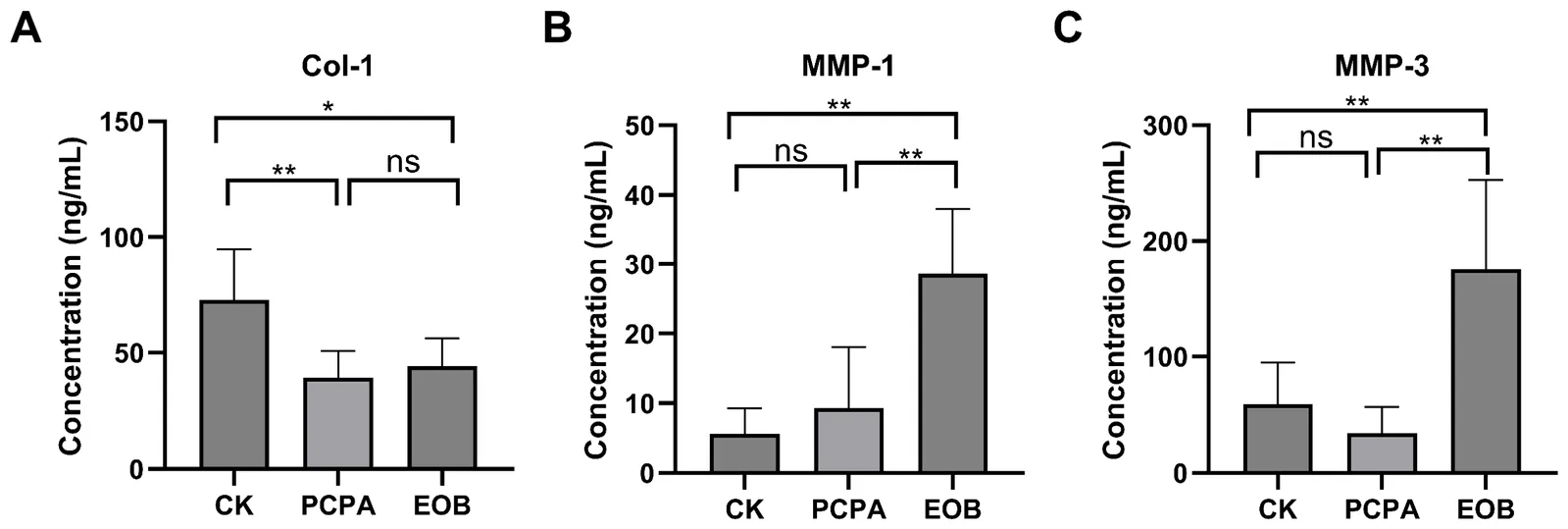
Effects of EOB on serum collagen and MMP levels in PCPA-treated mice. (**A**) Col-I, (**B**) MMP-1, (**C**) MMP-3. Data are mean ± SD. *n* = 10 per group. One-way ANOVA with Tukey’s post hoc test. * *p* < 0.05, ** *p* < 0.01; ns, not significant. Horizontal brackets denote pairwise comparisons vs. CK or PCPA group. All assays were performed using independent biological samples.

Compared with the CK group, serum Col-I concentration was significantly decreased in the PCPA group, whereas serum MMP-1 and MMP-3 levels showed no detectable alterations (Figure 6A–C). After EOB intervention, Col-I exhibited no statistically significant change relative to the PCPA group, while MMP-1 and MMP-3 were markedly upregulated. These observations indicate that EOB exerts divergent regulatory impacts on these circulating serum biomarkers.

Screening analyses identified several molecules with significant expression alterations upon EOB intervention, while their levels remained unaltered in PCPA-only model mice. Among these candidates, MMP-1 and MMP-3 were significantly upregulated following EOB treatment. Given their recognized biological roles in extracellular matrix (ECM) turnover, these two molecules were selected for subsequent in vitro functional exploration. In contrast, serum Col-I was not subjected to further cellular validation. Distinct from typical soluble signaling mediators, circulating Col-I mainly reflects systemic structural metabolic status and does not act as a fast-acting regulatory signaling molecule.

### 2.8. Cytotoxicity of Differentially Expressed Functional Molecules in Fibroblasts

**Figure 7:**
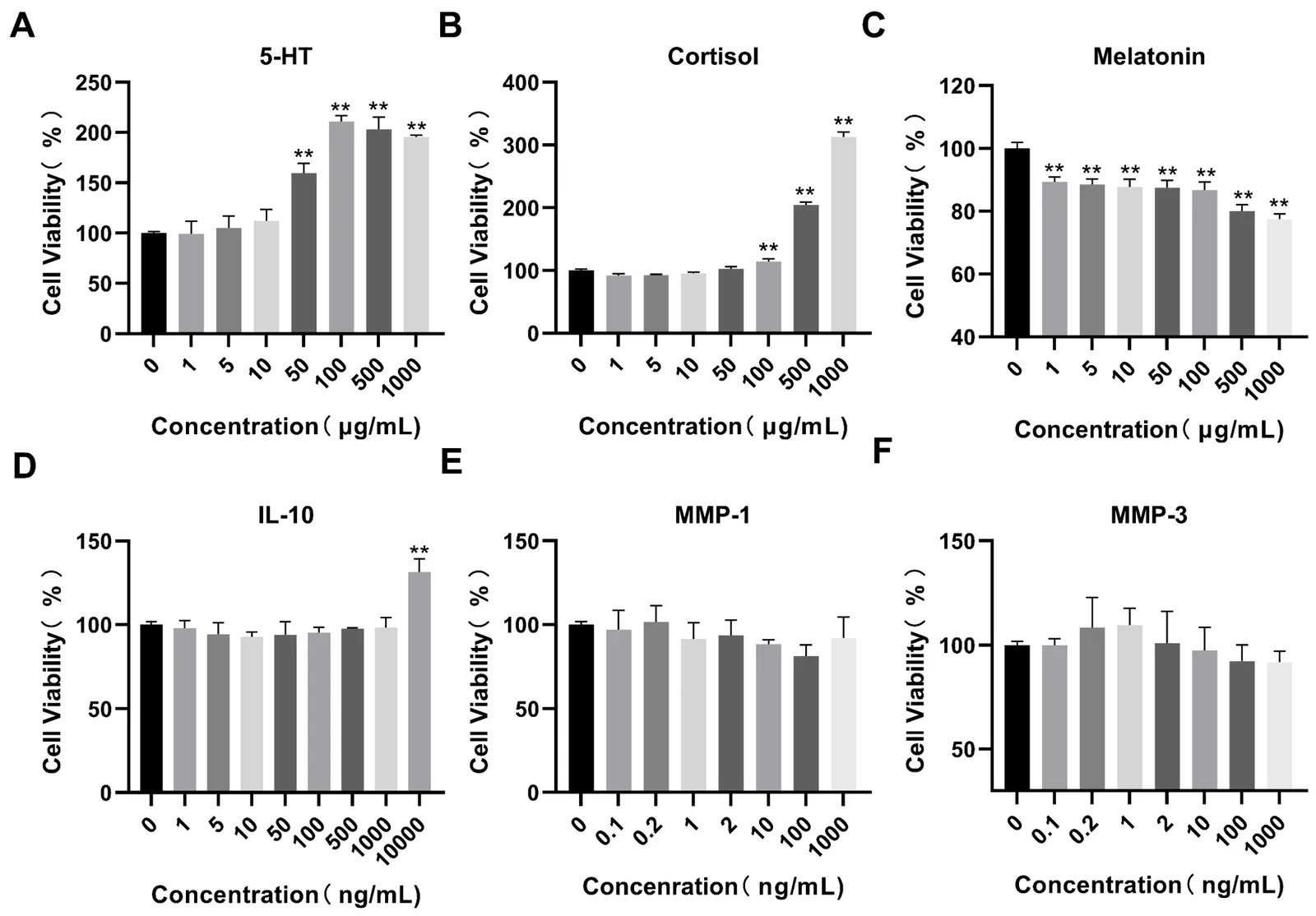
Effects of different molecules on fibroblast viability. (**A**) 5-HT, (**B**) Cortisol, (**C**) Melatonin, (**D**) IL-10, (**E**) MMP-1, (**F**) MMP-3. Results are from three independent biological experiments, with 3–4 technical replicate wells per experiment. One-way ANOVA with Tukey’s post hoc test. ** *p* < 0.01 vs. CK group.

Based on the in vivo findings described above (Section 2.5, Section 2.6 and Section 2.7), six circulating molecules exhibiting differential expression—5-HT, cortisol, melatonin, IL-10, MMP-1, and MMP-3—were selected for subsequent cellular experiments. Within the tested concentration ranges, these six molecules exerted no or only marginal cytotoxicity against dermal fibroblasts (Figure 7A–F). Low-, medium-, and high-concentration gradients were further chosen for each molecule to investigate their potential regulatory effects on dermal fibroblasts (Table 2).

The gradient concentrations used in this study were primarily applied to explore the dose-dependent effects of six mediators on fibroblasts in vitro and are therefore considered exploratory and supraphysiological. High, medium, and low concentrations were selected to comprehensively compare cellular responses across different intervention magnitudes. All working concentrations were screened according to the United States Pharmacopeia (USP) cytotoxicity evaluation standard to ensure non-cytotoxic conditions and eliminate experimental interference from cell death.

### 2.9. Functional Molecules Regulate Oxidative Stress Levels in Fibroblasts

**Figure 8:**
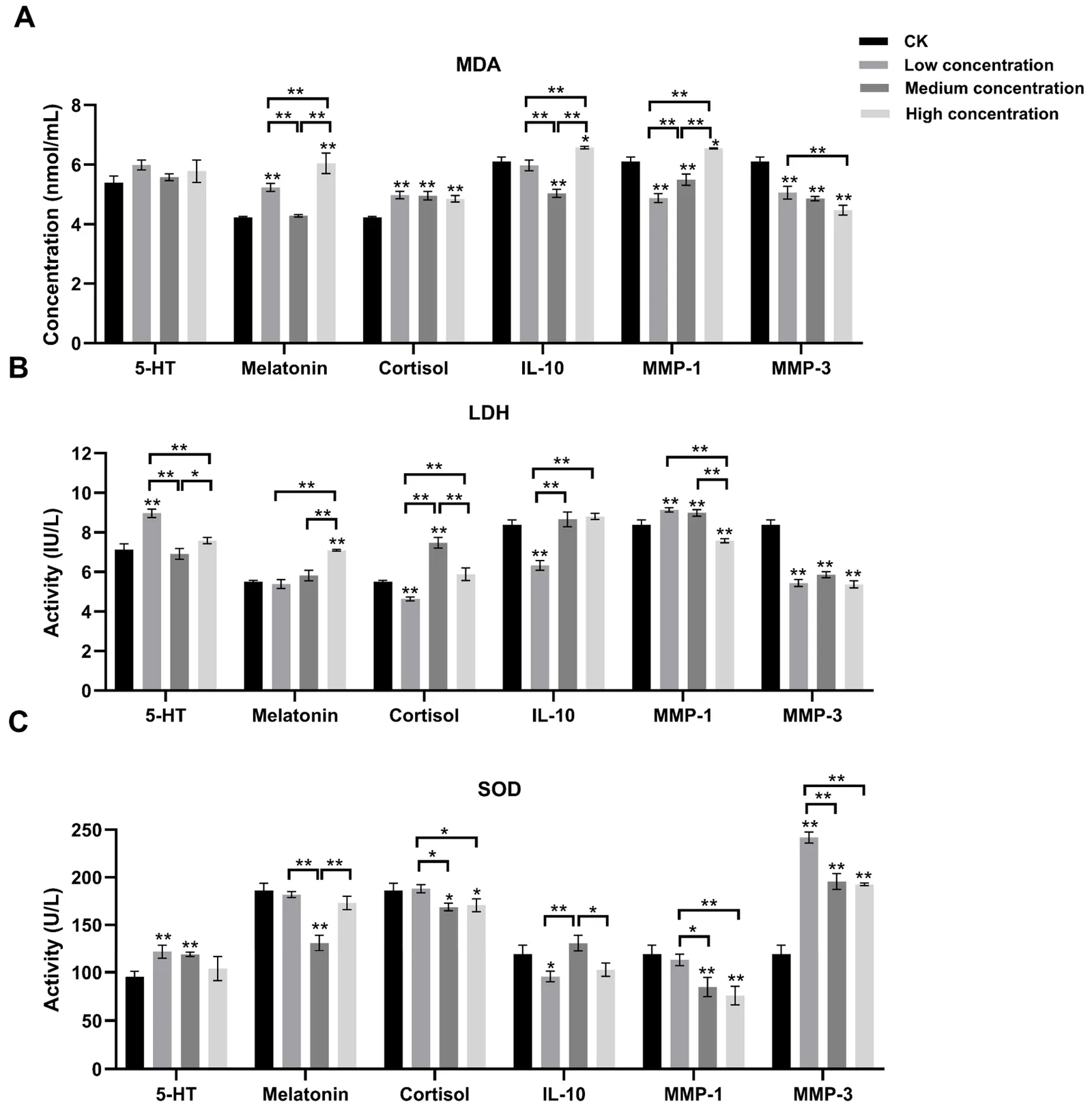
Effects of different molecules on oxidative stress levels in fibroblasts. (**A**) MDA, (**B**) LDH (**C**) SOD. Results are from three independent biological experiments, with 3–4 technical replicate wells per experiment. One-way ANOVA with Tukey’s post hoc test. * *p* < 0.05, ** *p* < 0.01 vs. CK group. Horizontal brackets denote pairwise comparisons among different-concentration groups for each molecule: * *p* < 0.05, ** *p* < 0.01.

At the tested concentrations, 5-HT exhibited no significant effect on cellular MDA levels. Cortisol markedly increased intracellular MDA accumulation. In contrast, melatonin induced elevated MDA generation at both low and high concentrations. IL-10 exerted a dual concentration-dependent effect on MDA production: medium-dose IL-10 suppressed MDA accumulation, whereas high-dose IL-10 promoted it. MMP-1 modulated MDA levels in a concentration-dependent manner: low-concentration MMP-1 significantly inhibited MDA generation, and this inhibitory effect gradually weakened with increasing concentration. Notably, at 10 ng/mL, MMP-1 reversed this trend and paradoxically upregulated cellular MDA levels. In contrast, MMP-3 consistently reduced MDA generation at all tested concentrations, with its inhibitory efficacy increasing in a concentration-dependent manner (Figure 8A).

Low-concentration 5-HT, high-concentration melatonin, and medium-concentration cortisol significantly increased extracellular LDH release; this effect was also observed at low-to-medium concentrations of MMP-1. In contrast, low-concentration cortisol, IL-10, high-concentration MMP-1, and MMP-3 at all tested concentrations markedly reduced extracellular LDH release (Figure 8B). 5-HT at low-to-medium concentrations and MMP-3 at all tested concentrations significantly enhanced SOD activity, whereas the remaining molecules inhibited SOD activity under specific concentration-dependent conditions (Figure 8C).

### 2.10. Functional Molecules Modulate Inflammatory Levels in Fibroblasts

**Figure 9:**
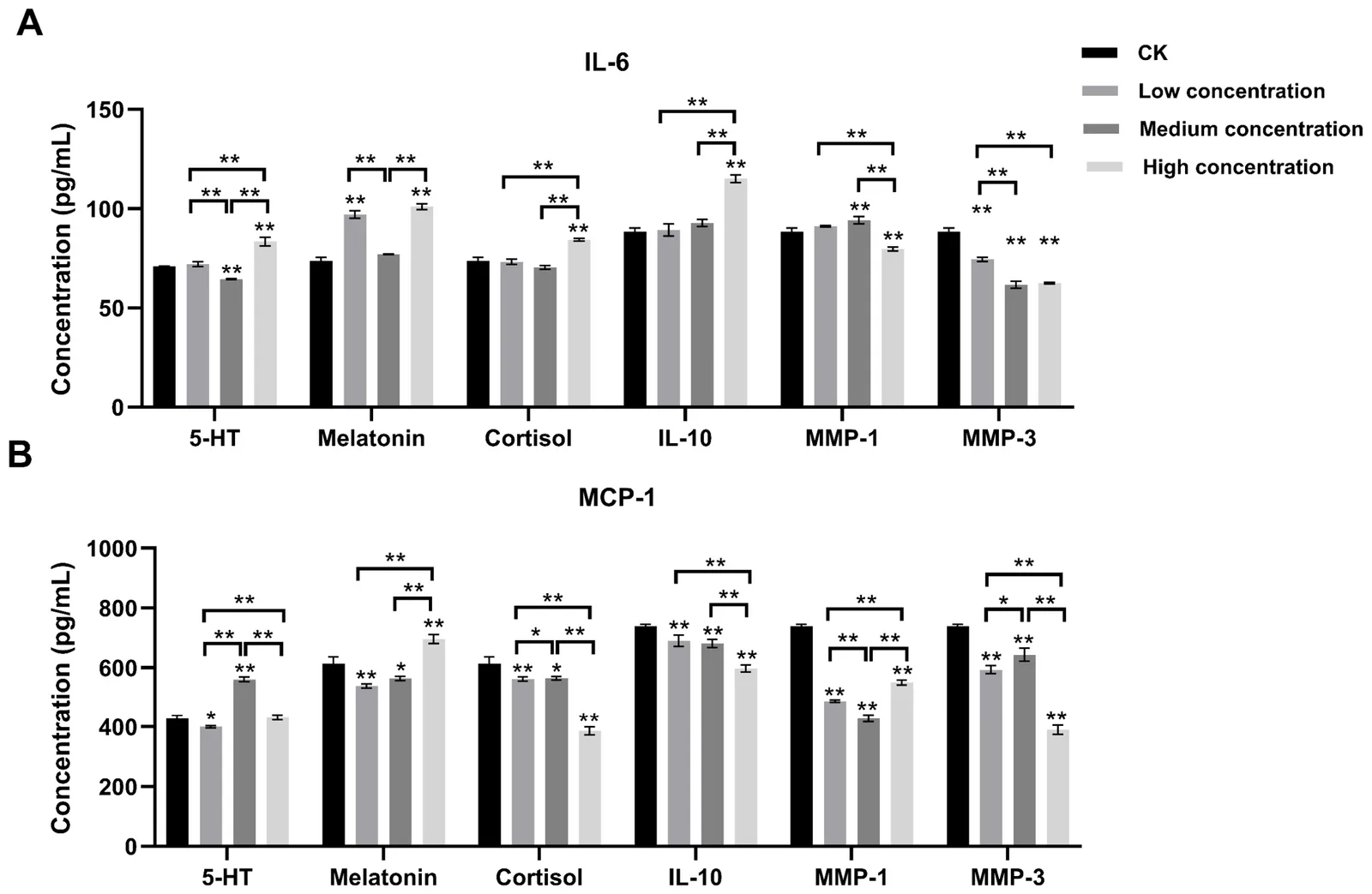
Effects of different molecules on inflammatory factor or chemokine levels in fibroblasts. (**A**) IL-6, (**B**) MCP-1. Results are from three independent biological experiments, with 3–4 technical replicate wells per experiment. One-way ANOVA with Tukey’s post hoc test. * *p* < 0.05, ** *p* < 0.01 vs. CK group. Horizontal brackets denote pairwise comparisons among different-concentration groups for each molecule: * *p* < 0.05, ** *p* < 0.01.

High concentrations of 5-HT, melatonin, cortisol, and IL-10 significantly promoted IL-6 production in fibroblasts. Low-concentration melatonin and medium-concentration MMP-1 also elevated cellular IL-6 levels, whereas MMP-3 significantly inhibited IL-6 secretion in a concentration-dependent manner (Figure 9A). Regarding MCP-1, medium-concentration 5-HT and high-concentration melatonin significantly upregulated its levels. Apart from these two treatment conditions, all other tested molecules decreased MCP-1 levels at all tested concentrations, and most of these changes reached statistical significance (Figure 9B).

### 2.11. Functional Molecules Alter ECM Synthesis and Secretion in Fibroblasts

**Figure 10:**
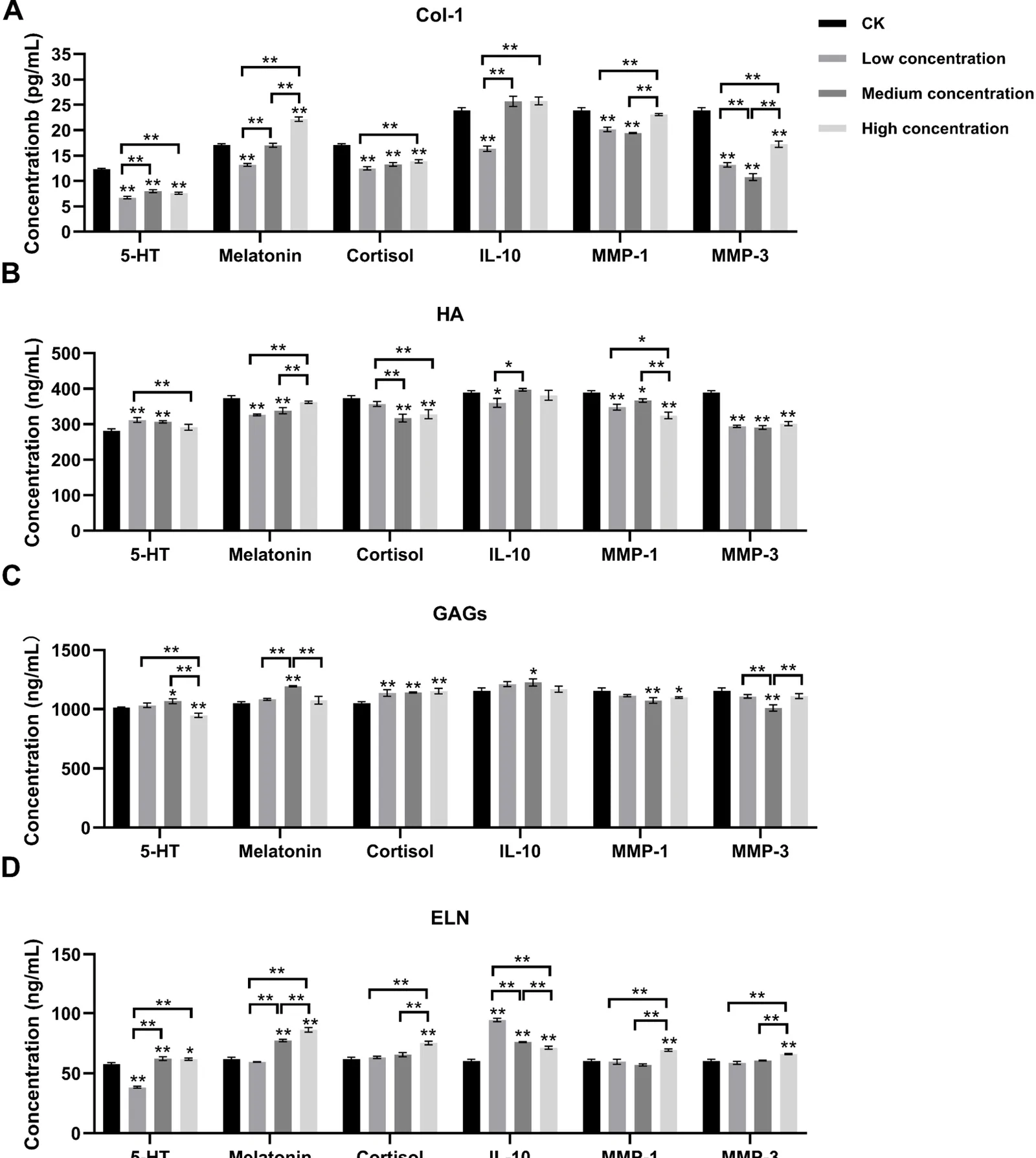
Effects of different molecules on ECM levels in fibroblasts. (**A**) Col-I, (**B**) HA, (**C**) GAGs, (**D**) Elastin. Results are from three independent biological experiments, with 3–4 technical replicate wells per experiment. One-way ANOVA with Tukey’s post hoc test. * *p* < 0.05, ** *p* < 0.01 vs. CK group. Horizontal brackets denote pairwise comparisons among different-concentration groups for each molecule, * *p* < 0.05, ** *p* < 0.01.

Different concentrations of the above functional molecules exhibited distinct regulatory effects on the levels of extracellular matrix components, including Col-I, HA, GAGs, and elastin, in dermal fibroblasts. Specifically, 5-HT, cortisol, MMP-1, and MMP-3 significantly reduced Col-I levels in cell lysates at all three tested concentrations. In contrast, high-concentration melatonin and medium-to-high concentrations of IL-10 increased Col-I abundance, with high-concentration melatonin showing the most pronounced effect (Figure 10A).

5-HT at low-to-medium concentrations elevated HA levels in cell lysates. In contrast, all other functional molecules decreased HA content to varying degrees (Figure 10B). Regarding GAG production, medium-concentration 5-HT, melatonin, and IL-10 markedly increased GAG levels. Similarly, cortisol elevated GAG levels across all tested concentrations. In contrast, MMP-1 and MMP-3 downregulated GAG levels, with significant inhibitory effects observed at the medium concentration (Figure 10C). Low-concentration 5-HT significantly reduced elastin levels, while other concentrations of 5-HT and all remaining tested molecules substantially promoted elastin production. The stimulatory effects of melatonin, cortisol, MMP-1, and MMP-3 were concentration-dependent, whereas IL-10 exerted the strongest pro-elastin effect at low concentrations (Figure 10D).

## 3. Discussion

Insomnia and anxiety form a mutually reinforcing pathological loop, and emotional disturbances further exacerbate skin damage, which is consistent with the established concepts of the brain–skin axis and psychodermatology [33,34,35,36]. This study investigated the ameliorative effects of EOB on an ICA mouse model and the underlying molecular mechanisms. PCPA inhibits central 5-HT synthesis, increases arousal, and disrupts sleep architecture, making it a widely used pharmacological agent for insomnia modeling [37]. The EPM is a classical paradigm for assessing anxiety-like phenotypes in rodents [38]. Behavioral results showed that PCPA significantly prolonged sleep latency and reduced open-arm activity in the EPM, confirming successful establishment of a stable ICA mouse model. EOB administration significantly reversed anxiety-like behaviors and alleviated sleep-disturbance-related behavioral phenotypes. In the pentobarbital-induced sedation test, EOB extended hypnotic duration from 21 min to 171 min, representing a 721.6% increase. However, given that this study relied solely on the righting-reflex behavioral assay without evaluating sleep architecture, this effect should be interpreted as pharmacodynamic potentiation with pentobarbital, rather than independent sleep-restoring bioactivity of EOB, and these findings also indicate a potential risk of excessive sedation.

5-HT, melatonin, and GABA are known to promote sleep [39,40], whereas cortisol levels are positively correlated with anxiety [41,42]. Neuroendocrine measurements revealed that PCPA significantly reduced central and peripheral 5-HT levels while elevating cortisol levels compared with the control group—a core mechanism underlying emotional and sleep disturbances. EOB treatment significantly reversed the 5-HT and cortisol imbalances and increased GABA levels, whereas melatonin showed only an increasing trend without statistical significance. These findings suggest that the 5-HT–cortisol axis is a key target through which EOB may modulate emotional homeostasis.

Psychological stress can induce skin inflammation and accelerate skin aging [9,43]. Type I and III collagen are core components maintaining skin structural integrity [44,45], and their loss is a hallmark of skin aging [46]. Cortisol inhibits collagen and hyaluronic acid synthesis [47], whereas melatonin exerts protective effects in stabilizing skin homeostasis [48,49]. Histological examination revealed no significant inflammatory infiltration in the skin of the ICA model, indicating that emotional stress-mediated skin damage primarily manifests as imbalanced molecular homeostasis and matrix metabolic disturbances rather than acute inflammatory lesions. Compared with the control group, PCPA significantly increased cutaneous cortisol levels while decreasing melatonin, type I collagen, and type III collagen levels, thereby disrupting skin molecular homeostasis. Relative to the PCPA group, EOB treatment effectively reversed these biochemical abnormalities: it significantly reduced cortisol and elevated melatonin, Col-I, and Col-III levels. These observations suggest that EOB exerts emotional-regulatory effects alongside beneficial effects on skin-associated molecular profiles, although no detectable recovery of skin tissue structure was observed histologically.

Circulating molecular analyses further provide insights into the systemic effects of EOB. EOB reversed PCPA-induced alterations in serum 5-HT, melatonin, and cortisol—findings consistent with previous reports [50,51,52]—and restored IL-10 levels, which may contribute to the relief of inflammation within the skin microenvironment [53]. The ICA model did not alter baseline MMP-1/3 levels but decreased Col-I expression, while EOB treatment upregulated MMP-1/3 while preserving collagen homeostasis. We speculate that this may represent an adaptive-remodeling phenotype characterized by elevated MMPs alongside improved molecular-level skin status. However, this interpretation remains speculative because we did not measure actual MMP enzymatic activity, TIMP abundance, or collagen-degradation products in this study. MMPs constitute a conserved family of zinc-dependent endopeptidases closely implicated in ECM remodeling and are capable of degrading nearly all ECM and basement membrane components [54,55]. Chronic emotional stress induces dermal matrix disorganization, abnormal matrix accumulation, and tissue stiffness. Hypothetically, moderate MMP upregulation may help clear disorganized pathological matrix and mitigate tissue sclerosis, while EOB stabilizes collagen synthesis and alleviates oxidative and inflammatory damage to counteract mild MMP-mediated matrix degradation. Therefore, MMPs may exert context-dependent bidirectional effects in stressed skin: excessive MMP activation causes tissue destruction, whereas moderate MMP elevation may facilitate microenvironmental remodeling. This proposed mechanism requires further experimental validation.

Based on the significantly altered circulating biomarkers in vivo, we selected six core molecules for in vitro functional validation in fibroblasts using non-cytotoxic concentrations. Multiple indicators covering oxidative stress (MDA, LDH, and SOD), inflammation (IL-6, MCP-1), and ECM metabolism (Col-I, HA, GAGs, and elastin) were detected as functional readouts. ROS-mediated oxidative stress impairs enzymes and cellular structures, aggravating skin inflammation and senescence [56]. SOD maintains cellular redox balance [57], while MDA and LDH are canonical markers of lipid peroxidation and cell membrane damage [58,59]. Excessive inflammation compromises skin barrier function [60]. As key inflammatory mediators, IL-6 and MCP-1 secreted by senescent fibroblasts sustain low-grade skin inflammation, and MCP-1 further recruits inflammatory cells and promotes tissue fibrosis [61,62,63]. Fibroblasts dominate dermal ECM biosynthesis and remodeling: Col-I was the major structural component of skin [64], elastin maintains skin elasticity [65], and GAGs stabilize the dermal microenvironment and support tissue regeneration [66]. Importantly, these in vitro fibroblast experiments only reflect cellular-level responsiveness to test molecules and cannot establish that these circulating molecules directly mediate in vivo skin outcomes. In vitro, the six molecules exhibited concentration-dependent, non-linear, differential effects: 5-HT and cortisol were predominantly detrimental, although low-dose cortisol activated Nrf2/HO-1 to reduce oxidative stress [67,68], whereas high-dose cortisol induced mitochondrial dysfunction and cell apoptosis [69]. By contrast, melatonin and IL-10 showed protective effects at optimal concentrations. In addition, although MMP-1/3 possess intrinsic ECM-degrading capability, they reduced oxidative stress and inflammatory responses in fibroblasts, further supporting the hypothetical adaptive protective role of MMPs under stress conditions. Overall, these circulating molecules may synergize or antagonize one another to regulate skin homeostasis. We hypothesize that EOB systematically remodels this circulating molecular network to improve emotion-skin homeostasis, and this network regulatory mechanism requires further in vivo verification.

Despite the informative findings, several methodological limitations warrant consideration when interpreting the present results. First, intraperitoneal delivery, while necessary to isolate systemic effects from direct skin contact or olfactory stimulation, does not replicate clinical aromatherapy routes and thus limits direct translational inference. Second, the sample size was determined based on published precedents and our preliminary data rather than formal power calculation—a pragmatic choice common in exploratory mechanistic studies. Third, sleep assessment relied on the righting-reflex assay, a well-validated behavioral measure of hypnotic activity, yet polysomnographic confirmation would be required to fully characterize sleep architecture. Fourth, the absence of EOB-only and positive control groups was a deliberate design decision to minimize animal use and focus on mechanistic dissection; however, this precludes full distinction between EOB’s intrinsic bioactivity and its pharmacodynamic interaction with pentobarbital. Fifth, the exclusive use of female mice was intended to reduce inter-individual variability, yet this inevitably restricts the generalizability of the conclusions. Sixth, skin evaluation was performed at the protein level, providing direct readouts of the molecules of interest, but histomorphological and genetic corroboration would add further robustness. Seventh, the in vitro concentrations were used for mechanistic proof-of-concept studies to explore dose-dependent cellular responses, rather than to precisely recapitulate in vivo local physiological mediator levels. These represent exploratory and supraphysiological conditions that may not faithfully reflect actual mediator concentrations in living tissues. Given that six distinct test substances were investigated, compound-specific published reference concentrations were unavailable in the published literature. This gradient-dosing approach is widely adopted in mechanistic proof-of-concept research. In addition, several compounds were dissolved in DMSO. While the uniform final DMSO concentration exhibited no cytotoxicity in preliminary tests, potential modulatory effects of DMSO on oxidative stress and inflammatory markers cannot be fully ruled out. This caveat should be considered when interpreting the in vitro results. Eighth, while we observed MMP-mediated adaptive remodeling, the upstream regulatory networks responsible for this response remain to be defined. Ninth, the relatively high EOB dose (300 mg/kg per injection) administered intraperitoneally was experimentally optimized to achieve stable systemic pharmacological effects. This dosing regimen differs from the low-dose, noninvasive application modes used in conventional essential oil aromatherapy and topical treatment, limiting direct clinical extrapolation of the present dose–effect findings. Notwithstanding these limitations, consistent trends observed across in vivo and in vitro datasets support the core inferences of this work. Future investigations addressing these caveats will further consolidate the mechanistic framework and translational potential of our findings.

## 4. Materials and Methods

### 4.1. Reagents

Para-chlorophenylalanine (PCPA, Cat. No. C6506) was purchased from Sigma-Aldrich, St. Louis, MO, USA.Gum acacia was obtained from Jonlnbio (Shanghai, China). 0.9% NaCl injection was purchased from Qitai (Qiqihar,, China). NaHCO_3_, Tween-80, 10% formalin fixative solution, hematoxylin, eosin staining solution, xylene, and ethanol were purchased from Sinopharm (Beijing, China). Phosphate-buffered saline (PBS) was purchased from Laibio (Shanghai, China). 4% paraformaldehyde (Cat. No. B46674) was purchased from Inno-chem (Beijing, China). ELISA kits for mice cortisol (Cat. No. YJ024317), 5-hydroxytryptamine (5-HT, Cat. No. YJ023354), γ-aminobutyric acid (GABA, Cat. No. M710087), type I collagen (Col-I, Cat. No. M710266), type III collagen (Col-III, Cat. No. C771521), matrix metalloproteinase-1 (MMP-1, Cat. No. MK2821B), matrix metalloproteinase-3 (MMP-3, Cat. No. MK2884A), interleukin-10 (IL-10, Cat. No. ml002285), superoxide dismutase (SOD, Cat. No. EJ1513267), and interleukin-6 (IL-6, Cat. No. P4004) were obtained from JonlnBio (Shanghai, China). ELISA kits for mice malondialdehyde (MDA, Cat. No. M709982), lactate dehydrogenase (LDH, Cat. No. M759395), monocyte chemoattractant protein-1 (MCP-1, Cat. No. P4025), hyaluronic acid (HA, Cat. No. YJ024992), glycosaminoglycan (GAG, Cat. No. YJ024456), and elastin (ELN, Cat. No. SB-EM4466) were purchased from Mlbio (Shanghai, China). High-purity monomer reagents of 5-HT (Cat. No. M66530), melatonin (Cat. No. M24580), and cortisol (Cat. No. M17805) for in vitro cell intervention were supplied by Meryer (Shanghai, China). Recombinant mouse IL-10 protein (Cat. No. NBL-286750, Chenhui Chuangju Biotechnology) was expressed in Pichia pastoris yeast, with >95% purity verified by SDS-PAGE and endotoxin level < 1 EU/μg determined by the LAL assay. Recombinant mouse MMP-1 (Cat. No. URPA097Mu02, Wuhan Yunclone Technology Co., Ltd., Wuhan, China) is of mouse origin and prokaryotically expressed in *Escherichia coli*. It carries an N-terminal His-tag, exhibits > 97% purity by SDS-PAGE, and has an endotoxin level < 1.0 EU/μg as quantified by the LAL assay. Recombinant mouse MMP-3 (Cat. No. CSB-EP014676MO, Wuhan Huamei Biological Engineering Co., Ltd., Wuhan, China), expressed in *Escherichia coli* from *Mus musculus*, is a full-length mature active protein with an N-terminal 6 × His-tag. It shows ≥90% purity by SDS-PAGE, Uniprot No. P28862, molecular weight of 46.2 kDa, and endotoxin level < 0.5 EU/μg. 4-Aminophenylmercuric acetate (APMA, Cat. No. P948559, Moligand™, ≥95% purity) was purchased from Aladdin Biochemical Technology Co., Ltd. (Shanghai, China). The Cell Counting Kit-8 (CCK-8, Cat. No. C8022-500TX6, Adamas-Life) was purchased from Solarbio (Beijing, China).

### 4.2. Essential Oil Blend Preparation

The essential oil blend (EOB) employed in this study consisted of lavender (*Lavandula angustifolia* Mill.), Kushui rose (*Rosa sertata* × *Rosa rugosa*), sweet orange (*Citrus sinensis* (L.) Osbeck), Roman chamomile (*Chamaemelum nobile* (L.) All.), clary sage (*Salvia sclarea* L.), and sandalwood (*Santalum album* L.) essential oils and was prepared by the R&D Center for Aromatic Plants of Shanghai Jiao Tong University. All constituent essential oils were obtained via hydrodistillation. The botanical origins and batch numbers of individual oils were as follows: lavender (batch No. LA-02-2024-B) and Roman chamomile (batch No. FF/24) were sourced from Xinjiang, China, and supplied by Shandong Freda Biotech Co., Ltd. ( Jinan, China); Kushui rose (batch No. ECO-14/06E) was sourced from Gansu, China; sweet orange (batch No. W3341, Italy), clary sage (batch No. 250503, Simiane-la-Rotonde, France), and sandalwood (batch No. SLFA/SWO/CO2/13/04/2026-27, India) were supplied by Yaqi Industrial (Shanghai) Co., Ltd. (Shanghai, China).

The EOB formula was designed based on the traditional Chinese medicinal “Monarch-Minister-Assistant-Guide (Jun-Chen-Zuo-Shi)” compatibility concept. This classical formulation strategy serves only as a theoretical rationale for component proportion design and formula optimization, not as experimentally validated functional evidence. The hypothesized roles and synergistic relationships of individual essential oils described below represent theoretical speculation for formula construction and do not constitute empirical conclusions generated by the present study.

Specifically, within this theoretical formulation framework, lavender oil (50%, *v*/*v*) was designated as the Monarch (Jun) component, putatively providing core sedative and anxiolytic potential to ameliorate ICA-associated behavioral abnormalities. Kushui rose oil (15%) and sweet orange oil (15%) served as Minister (Chen) components, presumed to assist in stabilizing emotional disturbances and reinforcing stress-alleviating effects. Roman chamomile oil (5%) and clary sage oil (5%) were assigned as Assistant (Zuo) components, hypothesized to exert auxiliary anti-inflammatory, antioxidant, and skin-protective properties against stress-induced cutaneous dysfunction. Sandalwood oil (10%) functioned as the Guide (Shi) component, theoretically proposed to harmonize the compatibility of the whole formula and facilitate the integrated pharmacological performance of the EOB. Notably, this study only characterizes the overall biological effects of the complete EOB formulation. This study cannot dissect the independent contribution of each individual essential oil or verify component-specific synergistic or antagonistic interactions.

### 4.3. Gas Chromatography–Mass Spectrometry (GC-MS) Analysis

The chemical components of EOB were identified using an Agilent 7890B-5977A GC-MS instrument. For sample pretreatment, the EOB sample was diluted 100-fold with an ethanol-n-hexane mixture (1:1, *v*/*v*), and the injection volume was 1 μL. An Agilent DB-WAX capillary column (30 m × 250 μm × 0.25 μm) was used for chromatographic separation. High-purity helium (99.999%) was used as the carrier gas with a constant flow rate of 1 mL/min, and the split ratio was set at 30:1. The injector temperature was maintained at 260 °C. The column temperature program was as follows: initial temperature held at 50 °C for 3 min, increased to 120 °C at 4 °C/min and held for 10 min, followed by a ramp to 220 °C at 2 °C/min with a final holding time of 2 min. Mass-spectrometry conditions were as follows: the electron ionization (EI) source was operated at 70 eV. The ion-source temperature and quadrupole temperature were set at 230 °C and 150 °C, respectively. Full-scan mode was used for mass acquisition. Compound identification was achieved by matching against the NIST14 mass-spectral library. The relative content of each volatile component was calculated using the peak-area normalization method.

### 4.4. Animals

Specific pathogen-free female BALB/c mice (9–11 weeks old, 18–22 g) were purchased from Shanghai Lingchang Biotechnology Co., Ltd. (Shanghai, China). All experimental animals were housed at the Laboratory Animal Center, Shanghai Jiao Tong University (Shanghai, China). The mice were maintained under standard laboratory conditions: a 12 h light/dark cycle, constant ambient temperature of 24 °C, and relative humidity of 50 ± 5%, with ad libitum access to standard chow and drinking water. Bedding was replaced daily, and all mice underwent a 1-week acclimatization period prior to formal experimentation.

All animal procedures were reviewed and approved by the Institutional Animal Ethics Committee of Shanghai Jiao Tong University (Approval No. A2024346-001). Animal handling and experimental operations were performed in strict accordance with the national guidelines for laboratory animal welfare and ethics. All experimental designs followed the 3R principles (Replacement, Reduction, Refinement). Appropriate humane endpoints were implemented throughout the experiment to minimize animal suffering and reduce the total number of experimental animals while ensuring valid and reproducible experimental results.

### 4.5. Preparation of Reagents

All solutions for intraperitoneal (i.p.) injection were freshly prepared on the day of administration to ensure chemical stability and experimental reproducibility. A 5% (*w*/*v*) sodium bicarbonate stock solution was prepared by dissolving sodium bicarbonate powder in warm ultrapure water. Subsequently, the stock solution was slowly titrated into sterile 0.9% saline, and pH was dynamically monitored using a pH meter (UnionTek, Beijing, China) to obtain weakly alkaline saline (pH 7.0–8.0). The prepared alkaline saline was temporarily stored at 4 °C and used within 24 h to prevent sample degradation.

To establish the ICA mouse model, PCPA powder was suspended in the above-mentioned weakly alkaline saline at a concentration of 500 mg/10 mL, with 10% gum arabic added as a suspending agent. The mixture was ultrasonicated for 15 min to form a homogeneous PCPA suspension (pH 7.0–8.0).

For EOB intervention, EOB was fully dissolved in 0.9% sterile saline containing 20 mg/mL Tween-80 (used as a non-toxic solubilizer) to prepare a 30 mg/mL EOB working solution. The EOB dosage and formulation were determined and verified in our preliminary exploratory experiments. In parallel, a matched vehicle-control solution containing only 20 mg/mL Tween-80 in 0.9% saline was prepared under identical conditions for vehicle-control injections.

### 4.6. Insomnia Mouse Model Establishment and Essential Oil Intervention

**Figure 11:**
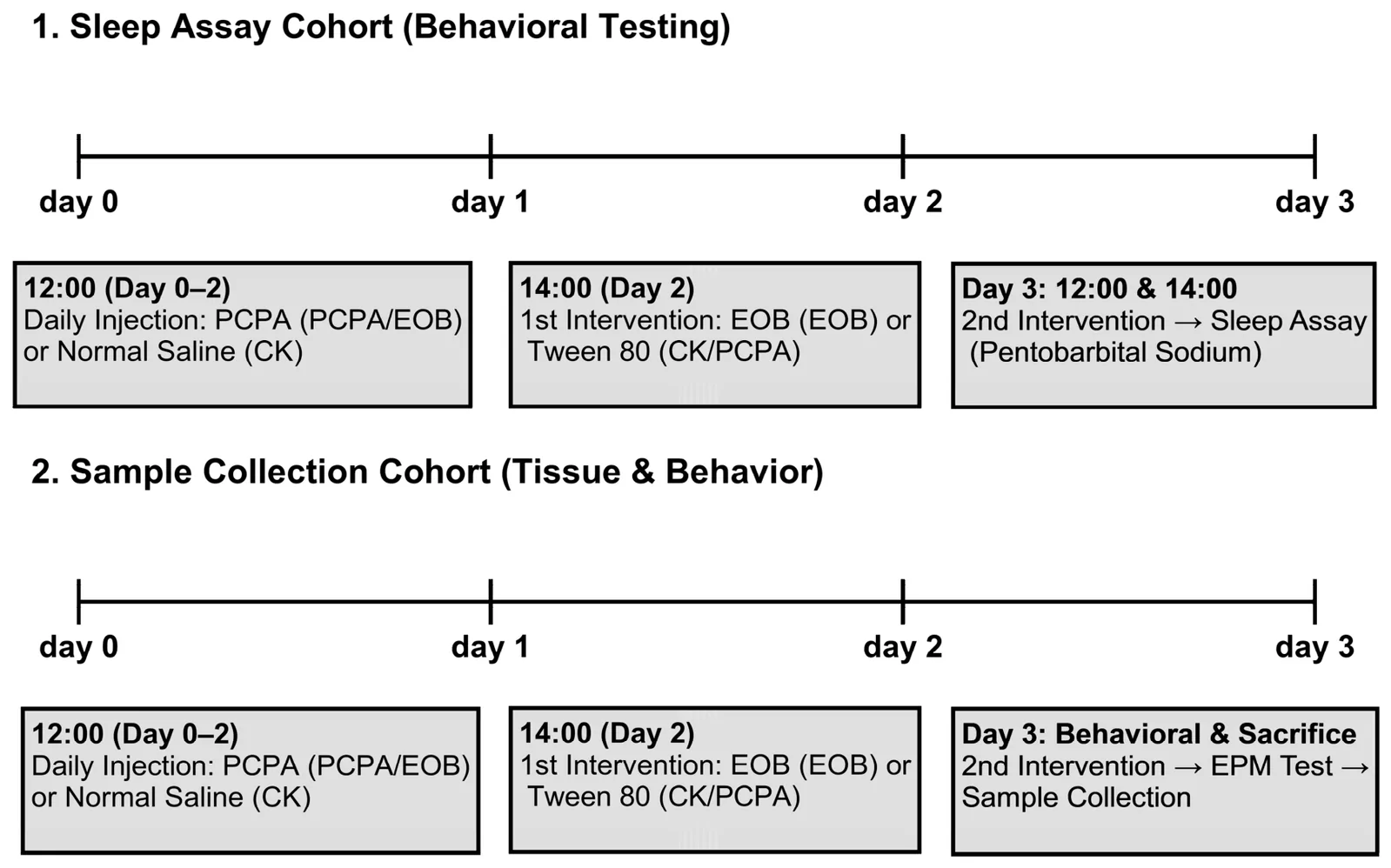
Schematic timeline of the experimental protocol. Forty-eight mice were randomly divided into two cohorts: Sleep Assay (*n* = 18) and Sample Collection (*n* = 30). Each cohort was further subdivided into three groups (*n* = 6/10 per group): Control (CK), ICA mouse model (PCPA), and EOB Intervention (EOB).

A total of 48 mice were used in this study. To avoid pentobarbital-related confounding effects, 18 mice were assigned to behavioral testing and 30 mice to sample collection (brain, serum, and skin). Grouping, modeling, and administration procedures were consistent between the two subsets (Figure 11). After one-week-long acclimatization, mice were weighed, numbered, and randomly divided into three groups (*n* = 6 or 10 per group): blank control (CK), ICA mouse model (PCPA), and EOB intervention.

The positive-control group was intentionally omitted in this experimental design. No clinically available drug can simultaneously regulate both systemic stress mediators and skin homeostasis. Classic anxiolytic/hypnotic agents mainly improve central emotional and sleep disturbances but exert minimal specific effects on cutaneous metabolism. Thus, this design ensured mechanistic specificity of EOB-derived emotion-skin regulation.

Six mice per group had their dorsal skin depilated prior to treatment. For ICA mouse modeling, PCPA suspension (1 mL/100 g) was intraperitoneally injected daily for three consecutive days; care was taken to avoid skin contact and olfactory stimulation. The CK group received an equal volume of weakly alkaline saline.

To exclude local skin and inhalation interference and specifically investigate circulation-mediated systemic effects, EOB was administered via intraperitoneal injection. At 2 h and 24 h after the final PCPA injection, EOB working solution (30 mg/mL, 300 mg/kg) was injected at 1 mL/100 g body weight. EOB was solubilized using 20 mg/mL Tween-80; complete dissolution was confirmed in preliminary tests [70]. Notably, this intraperitoneal administration was optimized for systemic mechanistic exploration and did not mimic clinical aromatherapy or topical application. No obvious toxicity or body-weight loss was observed, indicating acceptable in vivo tolerability. The CK and PCPA groups received equal volumes of Tween-80 vehicle solution.

### 4.7. Pentobarbital-Induced Sleep Test with Righting Reflex Assessment

For the sleep-test subgroup, 2 h after the final EOB administration, mice were intraperitoneally injected with sodium pentobarbital at 60 mg/kg, with dosage adjusted according to individual body weight. In accordance with commonly used behavioral evaluation criteria, sleep onset was defined as a continuous loss of the righting reflex for 10 consecutive seconds. Sleep latency (time from sodium pentobarbital injection to the loss of the righting reflex) and sleep duration (time from reflex loss to spontaneous reflex recovery) for each mouse were recorded to evaluate sleep-related behavioral phenotypes.

### 4.8. Elevated Plus Maze Test

The EPM test was conducted 30 min after the final EOB intervention in a quiet, dimly lit environment. All mice were acclimatized to the testing environment for 30 min prior to behavioral assessment to eliminate stress interference induced by environmental changes. Each mouse was gently placed in the central zone of the maze facing an open arm and allowed to freely explore the apparatus for 5 min. To avoid residual odor interference, the maze was thoroughly wiped with 75% ethanol after each test and air-dried before the next trial. All behavioral procedures were continuously recorded using a matched animal behavioral tracking system (Shxinruan, Shanghai, China). Behavioral parameters, including movement trajectory, open/closed arm entry times (OE/CE), open/closed arm residence time (OT/CT), and movement distance in open/closed arms (OD/CD), were quantitatively analyzed via Topscan 3.0 software (CleverSys, Reston, VA, USA). The percentages of open-arm entries, open-arm time, and open-arm movement distance were further calculated.

$$\mathrm{Percentage of open arm entries}\left(\%\right)=\frac{\mathrm{OE}}{\mathrm{OE}+\mathrm{CE}}\times 100\%$$

$$\mathrm{Percentage of time spent in open arms}\left(\%\right)=\frac{\mathrm{OT}}{\mathrm{OT}+\mathrm{CT}}\times 100\%$$

$$\mathrm{Percentage of distance travelled in open arms}\left(\%\right)=\frac{\mathrm{OD}}{\mathrm{OD}+\mathrm{CD}}\times 100\%$$

### 4.9. Collection and Processing of Serum, Brain Tissue, and Dorsal Skin

Three hours after the EPM test, mice were anesthetized via intraperitoneal injection of 2% sodium pentobarbital (40 mg/kg, dissolved in 0.9% saline). Orbital venous plexus blood sampling (0.5 mL) was performed under adequate anesthesia, followed by euthanasia via cervical dislocation. Brain tissues were rapidly dissected on ice, rinsed with pre-chilled PBS, weighed, and preserved at −80 °C for downstream biochemical detection. Whole-blood samples were kept at room temperature for 2 h, and then centrifuged at 3000 rpm, 4 °C for 15 min to harvest serum.

Brain tissues were homogenized in ice-cold PBS (1:9, *w*/*v*) using an ultrasonic cell disruptor (Jingxin, Shanghai, China). The homogenate was centrifuged at 3000 rpm, 4 °C for 15 min, and the resulting supernatant was collected.

Dorsal skin tissues were harvested from pre-depilated mice. Skin biopsies (10 mm and 8 mm diameter) were punched from identical anatomical positions using sterile biopsy punches (Tigergene, Nanjing, China). Subcutaneous fat and connective tissue were carefully trimmed away. The 10-mm skin specimens were homogenized in ice-cold PBS (1:9, *w*/*v*). After centrifugation (3000 rpm, 4 °C, 15 min), supernatants were collected. The 8-mm skin samples were weighed and fixed in 4% paraformaldehyde solution for subsequent H&E staining.

### 4.10. Brain Neurotransmitter and Hormone Levels Determination

The levels of 5-HT, melatonin, cortisol, and GABA in brain homogenate supernatants were determined using ELISA kits following the manufacturer’s instructions.

### 4.11. Skin Tissue H&E Staining

Intact 8-mm skin samples were subjected to H&E staining, observed, and photographed under a microscope. The pathological condition was scored according to criteria (Table 3), and the inflammation rate of each group was calculated as follows:

$$\mathrm{Skin inflammation rate}\left(\%\right)=\frac{\mathrm{number of mice with observable inflammatory cells}}{\mathrm{total number of mice}}\;\times 100\%$$

### 4.12. Skin Hormonal and Collagen Levels Determination

The levels of melatonin, cortisol, Col-I, and Col-III in the supernatant of skin homogenates were determined using ELISA kits following the manufacturer’s instructions.

### 4.13. Measurement of Serum Biomarker Levels and In Vitro Screening for Circulating Molecules

Serum levels of 5-HT, melatonin, cortisol, IL-10, Col-I, MMP-1, and MMP-3 were measured by ELISA. Circulating biomarker screening was performed based on a predefined, transparent selection strategy, and all statistical comparisons used a unified significance threshold of *p* < 0.05. Candidate molecules were selected as candidates if they were functionally involved in the neuroendocrine and inflammatory regulation of skin homeostasis, detectable in mice serum, and biologically active in dermal fibroblasts. The screening procedure integrated two comparative dimensions: pathological alterations induced by PCPA modeling and phenotypic variations responsive to EOB intervention. These ELISA kits specifically recognize soluble N-terminal propeptide fragments of collagen; these fragments accurately reflect systemic collagen turnover and dermal homeostatic status.

### 4.14. Cell Culture

Primary mouse skin fibroblasts (Cat. No. IMP-M089, Lot No. XM-20241106, Xiamen Immocell Biotechnology Co., Ltd., Xiamen, China) were derived from the dorsal dermal tissue of 6–8-week-old female BALB/c mice. All cellular experiments were performed using standardized primary fibroblast complete medium (Cat. No. IMP-M089-1, Immocell, China), consisting of 89% RPMI-1640 basal medium (Gibco, Waltham, MA, USA, Cat. No. 11875093), 10% fetal bovine serum (FBS, Gibco, Cat. No. 10099-141), and 1% penicillin-streptomycin mixture. To ensure stable cell biological activity and consistent experimental reproducibility, fibroblasts at passages 2–4 were used for all subsequent cellular interventions.

For in vitro pharmacological treatment, cells were seeded and cultured until they reached 80–90% confluence before drug administration. Cells were cultured in a humidified incubator maintained at 37 °C with 5% CO_2_ to support normal cell proliferation and physiological functions.

### 4.15. Cell Viability

The in vitro experimental groups included the blank group, the normal control group (CK), and compound treatment groups. Small-molecule substances (5-HT, cortisol, melatonin) were dissolved in DMSO to prepare stock solutions. To eliminate solvent interference, the final DMSO concentration in all culture systems was uniformly controlled at ≤1000 μg/mL. Preliminary cytotoxicity verification confirmed that this DMSO concentration exerted no observable adverse effects on fibroblast viability; thus, a dedicated DMSO vehicle control group was omitted in the formal experiment.

Recombinant proteins (IL-10, MMP-1, MMP-3) were reconstituted with sterile PBS strictly in accordance with the manufacturer’s guidelines. Before cellular treatment, pro-MMP-1 was pre-activated using 1 mM APMA at 37 °C for 1 h. Fibroblasts were collected and adjusted to a density of 5 × 10^4^ cells/mL, and then seeded in 96-well plates at a total volume of 100 μL per well (50 μL cell suspension + 50 μL gradient-concentration working solution). Each group was set with multiple replicate wells, and all cellular experiments were repeated in three independent biological trials with 2–3 technical replicates per trial to ensure experimental reliability.

The working concentrations of the six test substances were screened according to the USP cytotoxicity evaluation standard to select non-cytotoxic ranges. Given the multiple compounds under investigation, compound-specific published concentration data for each agent were unavailable. This strategy excluded cell-death-related interference on experimental outcomes. After 24 h of continuous incubation, the CCK-8 assay was performed to assess the effects of gradient concentrations of 5-HT, cortisol, melatonin, IL-10, MMP-1, and MMP-3 on fibroblast cell viability. The results were used to screen and determine the optimal low-, medium-, and high-intervention concentrations for subsequent mechanistic experiments. The CCK-8 assay was performed following the manufacturer’s official protocol, with minor, reasonable modifications adapted to the present cell experimental system.

### 4.16. Determination of Oxidative Stress, Inflammatory, and Extracellular Matrix Profiles in Fibroblasts

Cells were divided into the following groups: blank control group (CK), 5-HT group, cortisol group, melatonin group, IL-10 group, MMP-1 group, and MMP-3 group. Except for the CK group, three concentrations (low, medium, and high) were set for each drug-treated group. Mouse skin fibroblasts were harvested, adjusted to a density of 5 × 10^4^ cells/mL, and seeded into 6-well plates at 1 mL per well. Subsequently, 1 mL of test substance at the corresponding concentration was added to each well of the treatment groups, while 1 mL of complete medium was added to each well of the CK group. Three replicate wells were prepared for each group. Following 24 h of continuous incubation under standard culture conditions, cell culture supernatants were collected for LDH activity detection. Thereafter, adherent cells were harvested and lysed via repeated freeze–thaw cycles to fully release intracellular components. Cell lysates were centrifuged at 1000 rpm for 10 min, and the clarified supernatants were collected for subsequent biochemical detection.

The levels of LDH in culture supernatants, as well as SOD, MDA, IL-6, MCP-1, Col-I, HA, ELN, and GAG in cell lysates were quantitatively measured using commercial ELISA kits in strict accordance with the manufacturer’s protocols. To eliminate differences in cell loading and lysis efficiency, the total protein content of each cell lysate was determined via the BCA method. All intracellular biochemical indices were normalized to the corresponding total protein concentration for data normalization.

### 4.17. Statistical Analysis

All data were analyzed with IBM SPSS 24.0 and GraphPad Prism 10. Data are expressed as mean ± standard deviation (SD). Normality and homogeneity of variance were evaluated before statistical testing. One-way analysis of variance (ANOVA) with Tukey’s post hoc multiple-comparison test was performed for intergroup comparisons. Statistical significance was set at *p* < 0.05.

## 5. Conclusions

In conclusion, insomnia–anxiety stress disrupted cutaneous neuroendocrine homeostasis and ECM metabolism rather than inducing prominent tissue inflammation or structural damage. EOB intervention effectively improved stress-related behavioral abnormalities and partially remodeled skin molecular profiles at the biochemical level, with only subtle histological changes observed. In vitro fibroblast assays confirmed cellular sensitivity to circulating stress-related molecules, although these findings cannot fully confirm in vivo causal effects. Collectively, this study provides preliminary evidence supporting the emotion-skin regulatory effect of EOB via the potential brain–skin axis. Further mechanistic validation is required to elucidate the precise molecular pathways and molecular signaling mechanisms involved.

## Figures and Tables

**Table 1:** Effects on sleep status in mice.

Group	Sleep Latency (min)	Sleep Duration (min)
CK	5.5 ± 0.8	26.3 ± 5.6
PCPA	7.3 ± 0.8 **	20.9 ± 8.9
EOB	3.9 ± 0.6 ^##^	171.5 ± 9.3 ^##^

Data are mean ± SD, *n* = 6 per group. One-way ANOVA with Tukey’s post hoc test. ** *p* < 0.01 vs. CK group; ^##^ *p* < 0.01 vs. PCPA group.

**Table 2:** Concentrations of functional molecules used for fibroblast treatment.

Molecule	Low Concentration	Medium Concentration	High Concentration
5-HT	1 μg/mL	10 μg/mL	100 μg/mL
Cortisol	1 μg/mL	50 μg/mL	500 μg/mL
Melatonin	1 μg/mL	10 μg/mL	100 μg/mL
IL-10	0.1 ng/mL	10 ng/mL	10,000 ng/mL
MMP-1	0.1 ng/mL	0.2 ng/mL	10 ng/mL
MMP-3	0.1 ng/mL	1 ng/mL	1000 ng/mL

**Table 3:** Pathological condition scoring criteria.

Inflammatory Cell Infiltration	Tissue Edema Severity	Epidermal Structural Integrity	Score
No inflammatory cell infiltration, normal dermal tissue.	No interstitial edema, compact and regular tissue arrangement	Complete epidermal structure, clear and layered cell arrangement, no damage.	0
Sparse and scattered inflammatory cells, limited superficial dermal infiltration, negligible inflammation.	Mild interstitial edema, slight tissue gap widening without structural disorder.	Minor epidermal irregularity, intact basic structure, no obvious exfoliation or rupture.	1
Moderate inflammatory cell aggregation, diffuse infiltration in the dermis, obvious local inflammatory response.	Moderate tissue edema, significantly widened interstitial gaps, partial loose tissue arrangement.	Partial epidermal structural damage, local cell exfoliation and disorganized stratification.	2
Massive inflammatory cell infiltration, full-thickness dermal invasion, severe inflammatory lesion.	Severe tissue edema, extensively widened interstitial gaps, obvious tissue structural loosening.	Severe epidermal destruction, extensive exfoliation, blurred or lost layered structure.	3

This scoring system was used for blinded histopathological quantification of H&E-stained mouse skin. Three parameters—inflammatory infiltration, tissue edema, and epidermal integrity—were each scored 0–3 (four grades) and summed to a total pathology score (0–9), with higher scores indicating more severe damage. Five random microscopic fields per specimen were scored independently by two blinded investigators, followed by statistical analysis.

## Data Availability

The original contributions presented in this study are included in the article/supplementary material. Further inquiries can be directed to the corresponding author.

## Supplementary Materials

The following supporting information can be downloaded at: https://www.mdpi.com/article/10.3390/ph19101576/s1, Table S1: Composition of the essential oil blend (EOB) as determined by GC-MS; Report S1: Quality Test Report of Lavender Essential Oil; Report S2: Quality Test Report of Orange Essential Oil; Report S3: Quality Test Report of Clary sage Essential Oil; Report S4: Quality Test Report of Roman chamomile Essential Oil; Report S5: Quality Test Report of Kushui rose Essential Oil; Report S6: Quality Test Report of Sandalwood Essential Oil.

## Abbreviation

The following abbreviations are used in this manuscript:

5-HT	5-Hydroxytryptamine
APMA	4-aminophenylmercuric acetate
ANOVA	Analysis of Variance
CK	Blank Control
Col-I	Type I collagen
Col-III	Type III Collagen
ECM	Extracellular Matrix
ELISA	Enzyme-Linked Immunosorbent Assay
ELN	Elastin
EOB	Essential Oil Blend
EPM	Elevated Plus Maze
GABA	γ-Aminobutyric Acid
GAGs	Glycosaminoglycans
GC-MS	Gas chromatography–mass spectrometry
H&E	Hematoxylin and Eosin
HA	Hyaluronic Acid
ICA	Insomnia comorbid anxiety
i.p.	Intraperitoneal
IL-10	Interleukin-10
IL-6	Interleukin-6
LDH	Lactate Dehydrogenase
MCP-1	Monocyte Chemoattractant Protein-1
MDA	Malondialdehyde
MMP	Matrix Metalloproteinase
PBS	Phosphate-Buffered Saline
PCPA	para-Chlorophenylalanine
SD	Standard Deviation
SOD	Superoxide Dismutase
TNF	Tumor Necrosis Factor
USP	United States Pharmacopeia

## References

[1] Ditmer M., Gabryelska A., Turkiewicz S., Białasiewicz P., Małecka-Wojciesko E., Sochal M. (2021). Sleep Problems in Chronic Inflammatory Diseases: Prevalence, Treatment, and New Perspectives: A Narrative Review. J. Clin. Med..

[2] Maury E. (2019). Off the Clock: From Circadian Disruption to Metabolic Disease. Int. J. Mol. Sci..

[3] Steffey M.A., Scharf V.F., Risselada M., Buote N.J., Griffon D., Winter A.L., Zamprogno H. (2023). A Narrative Review of the Pathophysiology and Impacts of Insufficient and Disrupted Sleep. Can. Vet. J..

[4] Bhaskar S., Hemavathy D., Prasad S. (2016). Prevalence of Chronic Insomnia in Adult Patients and Its Correlation with Medical Comorbidities. J. Fam. Med. Prim. Care.

[5] Benjafield A.V., Sert Kuniyoshi F.H., Malhotra A., Martin J.L., Morin C.M., Maurer L.F., Cistulli P.A., Pépin J.-L., Wickwire E.M. (2025). Estimation of the Global Prevalence and Burden of Insomnia: A Systematic Literature Review-Based Analysis. Sleep Med. Rev..

[6] Everitt H., Baldwin D.S., Stuart B., Lipinska G., Mayers A., Malizia A.L., Manson C.C., Wilson S. (2018). Antidepressants for Insomnia in Adults. Cochrane Database Syst. Rev..

[7] Renoir T., Hasebe K., Gray L. (2013). Mind and Body: How the Health of the Body Impacts on Neuropsychiatry. Front. Pharmacol..

[8] Eldeiry D., Zandy M., Tayer-Shifman O.E., Kwan A., Marzouk S., Su J., Bingham K., Touma Z. (2020). Association between Depression and Anxiety with Skin and Musculoskeletal Clinical Phenotypes in Systemic Lupus Erythematosus. Rheumatology.

[9] Chen Y., Lyga J. (2014). Brain-Skin Connection: Stress, Inflammation and Skin Aging. Inflamm. Allergy Drug Targets.

[10] Jafferany M., Franca K. (2016). Psychodermatology: Basic Concepts. Acta Derm. Venereol..

[11] Gnatta J.R., Kurebayashi L.F.S., Turrini R.N.T., da Silva M.J.P. (2016). Aromatherapy and Nursing: Historical and Theoretical Conception. Rev. Esc. Enferm. USP.

[12] Fung T.K.H., Lau B.W.M., Ngai S.P.C., Tsang H.W.H. (2021). Therapeutic Effect and Mechanisms of Essential Oils in Mood Disorders: Interaction between the Nervous and Respiratory Systems. Int. J. Mol. Sci..

[13] Shaaban H.A.E., El-Ghorab A.H., Shibamoto T. (2012). Bioactivity of Essentisal Oils and Their Volatile Aroma Components: Review. J. Essent. Oil Res..

[14] Gong M., Dong H., Tang Y., Huang W., Lu F. (2020). Effects of Aromatherapy on Anxiety: A Meta-Analysis of Randomized Controlled Trials. J. Affect. Disord..

[15] Hines S., Steels E., Chang A., Gibbons K. (2018). Aromatherapy for Treatment of Postoperative Nausea and Vomiting. Cochrane Database Syst. Rev..

[16] Hwang E., Shin S. (2015). The Effects of Aromatherapy on Sleep Improvement: A Systematic Literature Review and Meta-Analysis. J. Altern. Complement. Med..

[17] Ebrahimi H., Mardani A., Basirinezhad M.H., Hamidzadeh A., Eskandari F. (2022). The Effects of Lavender and Chamomile Essential Oil Inhalation Aromatherapy on Depression, Anxiety and Stress in Older Community-Dwelling People: A Randomized Controlled Trial. Explore.

[18] Luo K., Ma X., Yao L., Li Y. (2018). Sedative and Hypnotic Effects of *Rosa sertata* × *Rosa rugosa* and *Rosa damascena* Essential Oils and Their Chemical Composition Analyzed by GC-MS. J. Shanghai Jiaotong Univ. Sci..

[19] Moner K. (2017). Lavender Essential Oil to Promote Sleep for People with Mood Disorders: A Qualitative Study. Ph.D. Dissertation.

[20] Ren Y.-L., Chu W.-W., Yang X.-W., Xin L., Gao J.-X., Yan G.-Z., Wang C., Chen Y.-N., Xie J.-F., Spruyt K. (2025). Lavender Improves Sleep through Olfactory Perception and GABAergic Neurons of the Central Amygdala. J. Ethnopharmacol..

[21] Son J.W., Kang P., Min S.S., Seol G.H. (2025). Differential Effects of Clary Sage (*Salvia sclarea* L.) Oil and Linalyl Acetate on Depression Levels in Diabetic Foot Ulcer Patients with T2DM: A Randomized Blinded Controlled Trial. Front. Med..

[22] Zhong Y., Zheng Q., Hu P., Huang X., Yang M., Ren G., Du Q., Luo J., Zhang K., Li J. (2019). Sedative and Hypnotic Effects of Compound Anshen Essential Oil Inhalation for Insomnia. BMC Complement. Altern. Med..

[23] Abdulazeez A.T., Musa M., Abubakar F.A., Saliu O.A., Tijjani H., Idowu O.A. (2025). Inhibitory Effect of Major Terpenoids of Essential Oil from *Agathosma betulina* Leaves on Collagenase and Elastase Enzymes Involved in Skin Aging. Pharm. Front..

[24] Kim B., Bae S. (2023). Evaluation of *Citrus junos* Essential Oil as a Promising Anti-Aging Ingredient: In Vitro and Clinical Studies on Skin Health Improvement. Asian J. Beauty Cosmetol..

[25] Kim D.Y., Won K.J., Kim Y.Y., Yoo D.Y., Lee H.M. (2024). Potential Wound Healing and Anti-Melanogenic Activities in Skin Cells of *Aralia elata* (Miq.) Seem. Flower Essential Oil and Its Chemical Composition. Pharmaceutics.

[26] Kürkçüoğlu M., Ağalar H.G., Temiz B., Duran A., Başer K. (2022). Chaerophyllum libanoticum Boiss. Et Kotschy: The Fruit Essential Oil, Composition, Skin-Whitening and Antioxidant Activities. Eur. J. Life Sci..

[27] Aremu O.O., Tata C.M., Sewani-Rusike C.R., Oyedeji A.O., Oyedeji O.O., Nkeh-Chungag B.N. (2019). Phytochemical Composition, and Analgesic and Anti-Inflammatory Properties of Essential Oil of *Chamaemelum nobile* (*Asteraceae* L. All) in Rodents. Trop. J. Pharm. Res..

[28] El Hachlafi N., Elbouzidi A., Batbat A., Taibi M., Jeddi M., Addi M., Naceiri Mrabti H., Fikri-Benbrahim K. (2024). Chemical Composition and Assessment of the Anti-Inflammatory, Antioxidant, Cytotoxic and Skin Enzyme Inhibitory Activities of *Citrus sinensis* (L.) Osbeck Essential Oil and Its Major Compound Limonene. Pharmaceuticals.

[29] Wijayadi L.J., Kelvin K. (2022). The Role of Essential Oils on Improving Elderly Skin Hydration. Sci. Midwifery.

[30] Samuelson R., Lobl M., Higgins S., Clarey D., Wysong A. (2020). The Effects of Lavender Essential Oil on Wound Healing: A Review of the Current Evidence. J. Altern. Complement. Med..

[31] Adewinogo S.O., Sharma R., Africa C.W.J., Marnewick J.L., Hussein A.A. (2021). Chemical Composition and Cosmeceutical Potential of the Essential Oil of *Oncosiphon suffruticosum* (L.) Källersjö. Plants.

[32] Feng X., Guo S., Wu Z., Lian H., Wu Y., Yang M., Fu B., Wang W. (2014). Evaluation of Relevant Information in Chronic Heart Failure Rats in Qi Deficiency and Blood Stasis Syndrome. China J. Tradit. Chin. Med. Pharm..

[33] Yoon K.-N., Chung J.H. (2025). Healthy Skin, Healthy Brain. J. Dermatol. Sci..

[34] Li J., Mao B., Tang X., Zhang Q., Zhao J., Zhang H., Chen W., Cui S. (2025). Endocrine and Metabolic Drivers of Sebum Dysregulation: Mechanisms and Therapeutic Strategies. Life Sci..

[35] Bobok N., Taskesen T. (2025). Stress-Induced Changes of the Skin: A Narrative Review. Cureus.

[36] Sharifi S., Gulati J., Shah V., Barr K., Jafferany M. (2026). Psychodermatology of the Female: Hormones, Skin and Mental Health Across the Lifespan. JEADV Clin. Pract..

[37] Rechtschaffen A., Lovell R.A., Freedman D.X., Whitehead W.E., Aldrich M., Barchas J., Usdin E. (1973). The Effect of Parachlorophenylalanine on Sleep in the Rat: Some Implications for the Serotonin-Sleep Hypothesis. Serotonin and Behavior.

[38] Carobrez A.P., Bertoglio L.J. (2005). Ethological and Temporal Analyses of Anxiety-Like Behavior: The Elevated Plus-Maze Model 20 Years on. Neurosci. Biobehav. Rev..

[39] Biso L., Bragazzi N.L., Bonaso M., Bersanetti E., Garbarino S., Scarselli M. (2025). Exogenous Melatonin as a Sleep-Promoting Agent Beyond Its Chronobiotic Properties: A Scoping Review of Its Effects on Key Sleep-Wake Neurotransmitters. Curr. Neuropharmacol..

[40] Siegel J.M. (2004). The Neurotransmitters of Sleep. J. Clin. Psychiatry.

[41] Chan I.I., Wu A.M.S. (2024). Assessing the Role of Cortisol in Anxiety, Major Depression, and Neuroticism: A Mendelian Randomization Study Using SERPINA6/SERPINA1 Variants. Biol. Psychiatry Glob. Open Sci..

[42] O’Donovan A., Hughes B.M., Slavich G.M., Lynch L., Cronin M.-T., O’Farrelly C., Malone K.M. (2010). Clinical Anxiety, Cortisol and Interleukin-6: Evidence for Specificity in Emotion–Biology Relationships. Brain Behav. Immun..

[43] Donato A., Belluzzi E., Venerando R., Ruggieri P., Vindigni V., Brun P. (2022). Skin Ageing and Inflammation: Role of Hyaluronan-Derived Anti-Inflammatory Molecules on Inflammatory Mediators’ Expression. Ital. J. Anat. Embryol..

[44] D’hondt S., Guillemyn B., Syx D., Symoens S., De Rycke R., Vanhoutte L., Toussaint W., Lambrecht B.N., De Paepe A., Keene D.R. (2018). Type III Collagen Affects Dermal and Vascular Collagen Fibrillogenesis and Tissue Integrity in a Mutant Col3a1 Transgenic Mice Model. Matrix Biol..

[45] Kisling A., Lust R.M., Katwa L.C. (2019). What Is the Role of Peptide Fragments of Collagen I and IV in Health and Disease?. Life Sci..

[46] Vitellaro-Zuccarello L., Garbelli R., Rossi V.D.P. (1992). Immunocytochemical Localization of Collagen Types I, III, IV, and Fibronectin in the Human Dermis: Modifications with Ageing. Cell Tissue Res..

[47] Choo J.H., Lee H.G., Lee S.Y., Kang N.G. (2023). *Iris pallida* Extract Alleviates Cortisol-Induced Decrease in Type 1 Collagen and Hyaluronic Acid Syntheses in Human Skin Cells. Curr. Issues Mol. Biol..

[48] Rusanova I., Martínez-Ruiz L., Florido J., Rodríguez-Santana C., Guerra-Librero A., Acuña-Castroviejo D., Escames G. (2019). Protective Effects of Melatonin on the Skin: Future Perspectives. Int. J. Mol. Sci..

[49] Slominski A., Kleszczyński K., Semak I., Janjetovic Z., Żmijewski M., Kim T.-K., Slominski R., Reiter R., Fischer T. (2014). Local Melatoninergic System as the Protector of Skin Integrity. Int. J. Mol. Sci..

[50] Golmohammadi M., Attari V.E., Salimi Y., Saed L., Nachvak S.M., Samadi M. (2025). The Effect of MIND Diet on Sleep Status, Mental Health, and Serum Level of BDNF in Overweight/Obese Diabetic Women with Insomnia: A Randomized Controlled Trial. Sci. Rep..

[51] Hasan S.M. (2022). Study on the Effect of Chamomile Extract on Melatonin Hormone Levels in Subjects Suffering from Insomnia and Anxiety. Biomedicine.

[52] Qian J., Zheng L., Huang M., Zhao M. (2024). Potential Mechanisms of Casein Hexapeptide YPVEPF on Stress-Induced Anxiety and Insomnia Mice and Its Molecular Effects and Key Active Structure. J. Agric. Food Chem..

[53] Kabat A.M., Pearce E.J. (2017). Inflammation by Way of Macrophage Metabolism. Science.

[54] Birkedal-Hansen H. (1995). Proteolytic Remodeling of Extracellular Matrix. Curr. Opin. Cell Biol..

[55] Kähäri V.-M., Saarialho-Kere U. (1999). Matrix Metalloproteinases and Their Inhibitors in Tumour Growth and Invasion. Ann. Med..

[56] Liu J., Tan F., Liu X., Yi R., Zhao X. (2020). Grape Skin Fermentation by *Lactobacillus fermentum* CQPC04 Has Anti-Oxidative Effects on Human Embryonic Kidney Cells and Apoptosis-Promoting Effects on Human Hepatoma Cells. RSC Adv..

[57] Berianidze K. (2014). Role of Redox- and Hormonal Metabolism in the Mechanisms of Skin Aging. Georgian Med. News.

[58] Bouzid M.A., Hammouda O., Matran R., Robin S., Fabre C. (2014). Changes in Oxidative Stress Markers and Biological Markers of Muscle Injury with Aging at Rest and in Response to an Exhaustive Exercise. PLoS ONE.

[59] Kaja S., Payne A.J., Naumchuk Y., Koulen P. (2017). Quantification of Lactate Dehydrogenase for Cell Viability Testing Using Cell Lines and Primary Cultured Astrocytes. Curr. Protoc. Toxicol..

[60] Jiang F., Wang S., Han D., Wei J., Wu Y., Liu Z. (2024). Glutamic-Acid Grafted Hyaluronic Acid Inhibits Inflammatory Factors via Fibroblast and Skin Model Tests. Skin. Res. Technol..

[61] Pereira B.I., Devine O.P., Vukmanovic-Stejic M., Chambers E.S., Subramanian P., Patel N., Virasami A., Sebire N.J., Kinsler V., Valdovinos A. (2019). Senescent Cells Evade Immune Clearance via HLA-E-Mediated NK and CD8+ T Cell Inhibition. Nat. Commun..

[62] Mansfield A.S., Nevala W.K., Dronca R.S., Leontovich A.A., Shuster L., Markovic S.N. (2012). Normal Ageing Is Associated with an Increase in Th2 Cells, MCP-1 (CCL1) and RANTES (CCL5), with Differences in sCD40L and PDGF-AA between Sexes. Clin. Exp. Immunol..

[63] Matsushita T., Hasegawa M., Hamaguchi Y., Takehara K., Sato S. (2006). Longitudinal Analysis of Serum Cytokine Concentrations in Systemic Sclerosis: Association of Interleukin 12 Elevation with Spontaneous Regression of Skin Sclerosis. J. Rheumatol..

[64] Kim M.-S., Song H.J., Lee S.H., Lee C.K. (2014). Comparative Study of Various Growth Factors and Cytokines on Type I Collagen and Hyaluronan Production in Human Dermal Fibroblasts. J. Cosmet. Dermatol..

[65] Procknow S.S., Kozel B.A. (2022). Emerging Mechanisms of Elastin Transcriptional Regulation. Am. J. Physiol. Cell Physiol..

[66] Yang P., Lu Y., Gou W., Qin Y., Tan J., Luo G., Zhang Q. (2024). Glycosaminoglycans’ Ability to Promote Wound Healing: From Native Living Macromolecules to Artificial Biomaterials. Adv. Sci..

[67] Li W. (2023). Effects of Cortisol on Oxidative Damage in Bovine Endometrial Epithelial Cells and the Protective Role of Selenium via the Nrf2 Signaling Pathway. Master’s Thesis.

[68] Zhong J., Wei Y., Yang C., Duan J., Guo L., Liu K., Dong J., Wang H., Li J., Cui L. (2025). Mechanism Study on the Effect of Cortisol on the Nrf2/HO-1 Pathway in Primary Bovine Endometrial Epithelial Cells. Chin. Vet. Sci..

[69] Liu Y., Sun Y., Wang S., Ma S., Yu W., Yin S., Wang T. (2024). Establishment of an In Vitro Culture Method for Intestinal Epithelial Cells of *Takifugu fasciatus* and the Effects of Cortisol on Their Physiological and Biochemical Characteristics. Prog. Fish. Sci..

[70] U.S. National Library of Medicine ChemIDplus-Advanced: Polyoxyethylene Sorbitan Monooleate (CAS No. 9005-65-6) (Database Retired December 2022; Data Migrated to PubChem). https://chem.nlm.nih.gov/chemidplus/.

