# Supplementary material for: Indirect Skin Homeostasis Modulation by an Essential Oil Blend in Mice with Insomnia Comorbid Anxiety: Sensitivity of Dermal Fibroblasts to Exogenously Applied Circulating Molecules
Source: Pharmaceuticals (Basel). 2026 Oct 4;19(10):1576. doi: 10.3390/ph19101576 (PMC13708607; doi:10.3390/ph19101576)
Supplement: Supplementary file 1 [file pharmaceuticals-19-01576-s001.zip › pharmaceuticals-4476911-supplementary.pdf]

# Indirect Skin Homeostasis Modulation by an Essential Oil Blend in Mice with Insomnia Comorbid Anxiety: Sensitivity of Dermal Fibroblasts to Exogenously Applied Circulating Molecules

## Supplementary Materials

Supplementary Table S1
Quality Test Report of Lavender Essential Oil
Quality Test Report of Orange Essential Oil
Quality Test Report of Clary sage Essential Oil
Quality Test Report of Roman chamomile Essential Oil
Quality Test Report of Kushui rose Essential Oil
Quality Test Report of Sandalwood Essential Oil

# Table of Contents

1. Supplementary Table S1. Composition of the essential oil blend (EOB) as determined by GC-MS.
2. Supplementary Report S1. Quality Test Report of Lavender Essential Oil
3. Supplementary Report S2. Quality Test Report of Orange Essential Oil
4. Supplementary Report S3. Quality Test Report of Clary sage Essential Oil
5. Supplementary Report S4. Quality Test Report of Roman chamomile Essential Oil
6. Supplementary Report S5. Quality Test Report of Kushui rose Essential Oil
7. Supplementary Report S6. Quality Test Report of Sandalwood Essential Oil

**Supplementary Table S1. Composition of the essential oil blend (EOB) as determined by GC-MS**

| No. | Compound | Retention times(min) | Retention index (RI) | Relative content (%) | Library (RI) |
|---|---|---|---|---|---|
| 1 | alpha-Pinene | 3.86 | 1014 | 0.77 | 1028±8 |
| 2 | 3-Thujene | 3.94 | 1018 | 0.05 | 1028±7 |
| 3 | Camphene | 4.67 | 1054 | 0.15 | 1071±8 |
| 4 | Isobutyl isobutyrate | 5.24 | 1082 | 0.14 | 1090±2 |
| 5 | beta-Pinene | 5.56 | 1098 | 0.11 | 1112±7 |
| 6 | Sabinene | 5.90 | 1111 | 0.13 | 1124±8 |
| 7 | 3-Carene | 6.61 | 1137 | 0.09 | 1147±7 |
| 8 | Myrcene | 7.04 | 1152 | 0.69 | 1161±7 |
| 9 | Isobutyl methacrylate | 7.13 | 1155 | 0.12 | 1168±6 |
| 10 | Isobutyl 2-methylbutyrate | 7.43 | 1166 | 0.09 | 1179±6 |
| 11 | 2-Methylallyl isobutyrate | 7.50 | 1169 | 0.04 | / |
| 12 | Limonene | 7.98 | 1186 | 13.67 | 1200±7 |
| 13 | beta-Phellandrene | 8.23 | 1195 | 0.14 | 1211±7 |
| 14 | Eucalyptol | 8.30 | 1198 | 0.52 | 1213±9 |
| 15 | Butyl butyrate | 8.63 | 1208 | 0.08 | 1220±8 |
| 16 | trans-beta-Ocimene | 9.13 | 1224 | 2.30 | 1250±6 |
| 17 | gamma-Terpinene | 9.40 | 1233 | 0.07 | 1246±9 |
| 18 | beta-Ocimene | 9.63 | 1240 | 0.65 | 1250±4 |
| 19 | 2-Methylallyl methacrylate | 9.74 | 1244 | 0.10 | / |
| 20 | p-Cymene | 10.07 | 1254 | 0.11 | 1272±8 |
| 21 | Hexyl acetate | 10.27 | 1261 | 0.43 | 1272±7 |
| 22 | Terpinolene | 10.51 | 1268 | 0.17 | 1283±7 |
| 23 | Isobutyl angelate | 10.78 | 1277 | 0.74 | 1290±3 |
| 24 | (E)-2-Methylbut-2-en-1-yl isobutyrate | 11.61 | 1303 | 0.08 | / |
| 25 | Butyl angelate | 12.52 | 1331 | 0.07 | 1417±N/A |
| 26 | Neoalloocimene | 13.38 | 1358 | 0.11 | / |
| 27 | 2-Butenoic acid, 2-methyl-, 2-methyl-2-propenyl ester, (Z)- | 13.43 | 1360 | 0.72 | / |
| 28 | 1-Octen-3-yl-acetate | 13.65 | 1366 | 1.40 | 1380±6 |
| 29 | 3-Methylpentyl methacrylate | 13.85 | 1372 | 0.18 | / |
| 30 | Pentan-2-yl 2-methylbut-2-enoate | 13.98 | 1377 | 0.42 | / |
| 31 | 2-Methylbutyl angelate | 14.12 | 1381 | 0.70 | 1395±N/A |
| 32 | (E)-2-Methylbut-2-en-1-yl methacrylate | 14.24 | 1385 | 0.08 | / |
| 33 | Hexyl butyrate | 14.84 | 1403 | 0.18 | 1414±8 |
| 34 | Linalool oxide A | 15.59 | 1427 | 0.16 | 1444±19 |
| 35 | 1-Octen-3-ol | 15.93 | 1438 | 0.25 | 1450±7 |

| No. | Compound | Retention times(min) | Retention index (RI) | Relative content (%) | Library (RI) |
|---|---|---|---|---|---|
| 36 | alpha-Copaene | 17.17 | 1477 | 0.11 | 1492±7 |
| 37 | 3-Methylpentyl angelate | 17.46 | 1486 | 0.77 | / |
| 38 | Camphor | 17.55 | 1489 | 0.13 | 1527±4 |
| 39 | (E)-2-Methyl-2-butenyl angelate | 17.75 | 1496 | 0.22 | / |
| 40 | Linalool | 18.88 | 1532 | 19.15 | 1547±7 |
| 41 | Linalyl acetate | 19.09 | 1539 | 27.14 | 1555±10 |
| 42 | Pinocarvone | 19.13 | 1541 | 0.18 | / |
| 43 | alpha-Santalene | 19.58 | 1555 | 0.24 | 1575±9 |
| 44 | trans-alpha-Bergamotene | 19.99 | 1569 | 0.08 | 1580±6 |
| 45 | beta-Caryophyllene | 20.18 | 1575 | 1.60 | 1595±16 |
| 46 | Terpinen-4-ol | 20.36 | 1581 | 1.58 | 1602±9 |
| 47 | Lavandulyl acetate | 20.58 | 1588 | 2.83 | / |
| 48 | Alloaromadendrene | 21.66 | 1619 | 0.46 | 1650±11 |
| 49 | (-)-trans-Pinocarveol | 21.94 | 1627 | 0.23 | 1664±7 |
| 50 | (E)-beta-Famesene | 22.60 | 1644 | 0.48 | 1664±6 |
| 51 | Lavandulol | 22.90 | 1652 | 0.29 | 1677±9 ( |
| 52 | alpha-Terpineol | 23.62 | 1671 | 0.93 | 1697±10 |
| 53 | Borneol | 23.71 | 1673 | 0.86 | 1702±15 |
| 54 | Isodaucene | 24.73 | 1700 | 0.14 | / |
| 55 | Neryl acetate | 24.92 | 1703 | 0.53 | 1724±9 |
| 56 | (+)-delta-Cadinene | 26.23 | 1726 | 0.13 | 1758±13 |
| 57 | Geranyl acetate | 26.41 | 1729 | 0.61 | 1752±11 |
| 58 | Citronellol | 27.17 | 1742 | 6.16 | 1765±9 |
| 59 | Nerol | 28.96 | 1774 | 0.65 | 1797±11 |
| 60 | 2-Tridecanone | 29.26 | 1779 | 0.25 | 1809±6 |
| 61 | Geraniol | 32.28 | 1827 | 3.37 | 1847±10 |
| 62 | Caryophyllene oxide | 38.93 | 1930 | 0.13 | 1989±19 |
| 63 | Methyleugenol | 41.77 | 1977 | 0.13 | 2013±16 |
| 64 | Heneicosane | 48.15 | 2094 | 0.10 | 2100 |
| 65 | Eugenol | 49.29 | 2117 | 0.14 | 2169±16 |
| 66 | tau-Cadinol | 49.70 | 2125 | 0.13 | 2169±16 |
| 67 | Ylangenal | 55.06 | 2235 | 0.48 | / |
| 68 | Tricosane | 57.61 | 2290 | 0.18 | 2300 |
| 69 | alpha-Santalol | 58.08 | 2300 | 1.30 | 2350 |
| 70 | Bergamotol, Z-alpha-trans- | 58.61 | 2313 | 0.12 | 2328±N/A |
| 71 | Farnesol | 58.99 | 2321 | 0.44 | 2350±8 |
| 72 | beta-Santalol | 61.55 | 2380 | 0.57 | 2413±22 |

- **Supplementary Report S1. Quality Test Report of Lavender Essential Oil**

***CERTIFICATE OF ANALYSIS AND QUALITY***

**Product name: Lavender oil organic lot# LA-02-2024-B**
**INCI name: Lavandula Angustifolia**
**Product code: CAS 8000-28-0**
**Manufacturing date: 22JUL/2024**
**Expiry date: 22/JUL/2028**

| | |
|---|---|
| APPEARANCE | COLOURLESS TRANSPARENT LIQUID |
| RELATIVE DENSITY AT 20C | 0.881 |
| REFRACTIVE INDEX AT 20C | 1.461 |
| OPTICAL ROTATION | - 8.50 |
| COLOUR | PALE YELLOW |
| ODOUR | CHARACTERISTIC |

CONTENT OF TIPICAL INGREDIENT, %
(By Gas-Chromatography)

| | | MIN % | MAX % |
|---|---|---|---|
| LINALOOL | 31.03 | 25.0 | 34.0 |
| LINALYLACETATE | 27.82 | 25.0 | 42.0 |
| LAVANDULILACETATE | 3.22 | 2.0 | 5.0 |
| CAMPHOR | 0.26 | - | 0.6 |
| LAVANDULOL | 1.02 | 0.3 | |
| 1.8 CINEOL | 0.88 | - | 2.0 |
| TERPINEN 4-OL | 4.80 | 2.0 | 5.0 |
| TRANS-BETA-OCIMENE | 2.95 | 2.0 | 5.0 |
| ALFA TERPINEOL | 1.29 | 0.8 | 2.0 |
| CIS-BETA-OCIMENE | 5.94 | 3.0 | 9.0 |
| 3 OKTANONE | 1.41 | 0.2 | 1.6 |
| LIMONENE | 0.38 | - | 0.6 |
| BETA-PHELLANDRENE | 0.13 | | |

Note: 1.8-cineole and beta-phellandrene are often coeluted.

ECOARTS

- **Supplementary Report S2. Quality Test Report of Orange Essential Oil**

## ANALYSIS CERTIFICATE

| | |
|---|---|
| Product: | Orange Oil |
| Sample: | W3341 |
| Shelf Life (at 4°C under Nitrogen) : | 24 months |
| Shelf Life (at 25°C under Nitrogen): | 12 months |

| ***Analysis*** | ***Value*** |
|---|---|
| Appearance: | Yellow Reddish / Giallo con tono Rosso |
| Flavour: | Fresh, typical of fruit |
| Optical Rotation at 25°C : | 97,49 |
| Refractive Index at 20°C : | 1,473 |
| Specific Gravity at 20°C : | 0,850 |
| Peroxide Value meq / kg / N° di Perossidi: | 8,430 |
| Alpha Pinene Content % GC: | 0,572 |
| Myrcene Content % GC: | 1,994 |
| Octanal Content% GC: | 0,326 |
| D-Limonene Content% GC: | 95,036 |
| Linalool Content% GC: | 0,318 |
| Decanal Content% GC: | 0,289 |
| Aldehydes Content% GC: | 0,828 |

**Catania 29/01/2026**

ECOARTS

- **Supplementary Report S3. Quality Test Report of Clary sage Essential Oil**

| Certificat d'analyse / Analysis certificate | |
|---|---|
| Huile essentielle / Essential oil : | Sauge sclarée / Clary sage |
| Lot / Batch : | 250503 |
| *Date début essai / Analysis start date :* | *30/10/2025* |
| *Date fin essai / Analysis end date :* | *03/11/2025* |
| *Date fabrication / Manufacture date :* | *30/07/2025* |
| *DLUO / Expiry date :* | *30/07/2028* |

| Essai / Test | Résultat / Result |
|---|---|
| INDICE DE REFRACTION | 1,461 |
| POUVOIR ROTATOIRE° | -19,00 |
| LINALOOL | 18,22% |
| Alpha-TERPINEOL | 0,72% |
| LINALYL ACETATE | 64,09% |
| GERMACENE D | 5,11% |
| SCLAREOL | 0,20% |

**Simiane la Rotonde,** **04/11/2025**

ECOARTS

- **Supplementary Report S4. Quality Test Report of Roman chamomile Essential Oil**

*Confidential to recipient* *Date: 01/10/2024*

**CERTIFICATE OF ANALYSIS**

**Product Description:** **ENGLISH CHAMOMILE OIL (ANTHEMIS NOBILIS)**
**Lot / Batch:** **FF/24**

**Cropping date:** **1 August 2024**

**Date of expiry:** **1 August 2027 (if stored correctly)**

| TEST | RESULTS |
|---|---|
| COLOUR | PALE BLUE / GREEN |
| ODOUR | FRESH FRUITY HERBACEOUS |
| APPEARANCE | MOBILE LIQUID |
| REFRACTIVE INDEX@ 20˚C | 1.4421 |
| SPECIFIC GRAVITY @ 20˚C | 0.9022 |
| OPTICAL ROTATION @ 20˚C | + 3.7˚C |
| FLASH POINT ( closed cup method) | >64ºC |
| ESTER VALUE | 75% |
| ACID VALUE | 5 mg /g |

ECOARTS

- **Supplementary Report S5. Quality Test Report of Kushui rose Essential Oil**

# Certificate Of Analysis（COA）

**Product Name:** Shuiku Rose Essential Oil
**INCI Name:** Rosa Rugosa Flower Oi
**Material Code:** 1.1.086.48.012
**Batch No.:** ECO-24/06E

**Production Process:** Distillation
**Extracted Part:** Flower
**Production Date:** June 2024
**Shelf-life:** 5 years

| Items | Specification | Result |
|---|---|---|
| **Appearance** | Pale yellow to light-yellow clear free-flowing liqui | Complies |
| **Odor** | Strong floral aroma of Kushui rose | Complies |
| **Relative Density (25 °C)** | 0.8560 ~ 0.900 | Complies |
| **Refractive Index (25 °C)** | 1.4600 ~ 1.4730 | Complies |

**Main Components and Contents (%) [GC-MS]**

| | | |
|---|---|---|
| Citronellol | 40.0 ~ 54.0 | 43.08 |
| Geraniol | 7.0 ~ 18.0 | 11.44 |
| Linalool | 1.0 ~ 4.0 | 2.25 |
| Nerol | 2.0 ~ 6.0 | 3.08 |
| Citronellyl acetate | 2.0 ~ 4.5 | 4.30 |

- **Supplementary Report S6. Quality Test Report of Sandalwood Essential Oil**

## CERTIFICATE OF ANALYSIS

PRINTED ON : 22.04.2026

**Item Code : SLFA03S001** **Batch No. : SLFA/SWO/CO2/13/04/2026-27**

**Item Name : SANDALWOOD OIL CO2**

| | |
|---|---|
| **CAS #** | 8006-87-9 |
| **EINECS #** | 284-111-1 |
| **Botanical Name** | Santalum album |
| **Product Description** | Clear, pale yellow to Golden color Liquid having pleasant , sweet, woody and Balsamic odor |
| **Origin** | Obtained by Supercritical Fluid Extraction sourced from South Indian plantations |
| **Plant Part Used** | Logs |
| **Manufacturing Date** | April , 2026 |
| **Expiry Date** | March,2029 |
| **Blend** | 100 % Pure natural and no additives are added |

| SR. NO. | PARAMETERS | SPECIFICATION | RESULT | PROTOCOL / METHOD |
|---|---|---|---|---|
| | ORGANOLEPTIC CHARACTERISTICS | | | |
| 1 | Appearance | Clear, pale yellow to Golden Color Liquid | CONFORMS | Visual |
| 2 | Odor | pleasant , sweet, woody and Balsamic | CONFORMS | Organoleptic |
| 3 | Taste | Characteristic | CONFORMS | Organoleptic |
| | PHYSICO-CHEMICAL SPECIFICATIIONS | | | |
| 4 | Refractive Index @ 25°C | 1.5000 - 1.5100 | 1.5073 | In House Method |
| 5 | Specific Gravity @ 25°C | 0.9650 - 0.9850 | 0.9833 | In House Method |
| 6 | Optical Rotation | -20° to -15° | -20 to – 15 % | In House Method |
| 7 | Solubility | Insoluble in water, Soluble in Alcohol | CONFORMS | <USP 561> |
| 8 | Esters, calculated as santalyl acetate | NMT 7 % | < 7 % | GCMS |
| 9 | Main Components | Alpha-Santalol: 41.0-55.0%<br>Beta-Santalol: 16.0-24.0%<br>Total Santalol: > 90 % | 53.33 %<br>23.31 %<br>90.73 % | GCMS |
| | HEAVY METALS | | | |
| 10 | Lead | NMT 0.5 PPM | CONFORMS | AOAC $16^{TH}$ Edition |
| 11 | Arsenic | NMT 0.1 PPM | CONFORMS | AOAC $16^{TH}$ Edition |
| 12 | Cadmium | NMT 0.5 PPM | CONFORMS | AOAC $16^{TH}$ Edition |
| 13 | Mercury | NMT 0.1 PPM | CONFORMS | AOAC $16^{TH}$ Edition |
| 14 | PESTISIDES | As per EU 396 | COMPLIES | |

ECOARTS

| | | | | |
|---|---|---|---|---|
| | ***MICROBIOLOGICAL PROFILE*** | | | |
| 15 | **Total Plate Count** | ***< 1000 cfu/g*** | ***CONFORMS*** | ***BAM Online*** |
| 16 | **Yeast / Moulds /Fungi** | ***< 100 cfu/g*** | ***CONFORMS*** | ***BAM Online*** |
| 17 | **E-Coli** | ***ABSENT/g*** | ***CONFORMS*** | ***BAM Online*** |
| 18 | **Salmonella** | ***ABSENT in 25 gms*** | ***CONFORMS*** | ***BAM Online*** |
| 19 | **Coliforms** | ***< 10 cfu/g*** | ***CONFORMS*** | ***BAM Online*** |
| 20 | **P. Aeruglinosa** | ***ABSENT*** | ***CONFORMS*** | ***BAM Online*** |
| 21 | **S. Aureus** | ***ABSENT*** | ***CONFORMS*** | ***BAM Online*** |
| 22 | **Staphylococcus sp** | ***ABSENT in 10 g*** | ***ABSENT*** | ***BAM Online*** |
| | **MYCOTOXINS** | | | |
| 23 | **Aflatoxin B1** | ***<5 ppb*** | ***CONFORMS*** | ***ASTA 24.2. 1997 Edition*** |
| 24 | **Aflatoxin (B1+B2+G1+G2)** | ***<10ppb*** | ***CONFORMS*** | ***ASTA 24.2. 1997 Edition*** |

---

| | |
|---|---|
| **Legislation** | Natural |
| **Stability** | Avoid strong acids strong bases and all oxidizing agents. |
| **Uses** | Flavors & Fragrance |
| **Handling Precaution** | Handle in adequate ventilation, avoid skin and eye contact, use goggles or face shield and wear protective clothing |
| **Packing & Storage** | Store at ambient temperature, dry and well-ventilated area, away from heat source and protected from light. Store in tightly sealed original containers, packed in HDPE Food grade cans. |
| **Labeling** | The Label displays Product Name, Batch No., Net Weight, Gross Weight and other information / marks specified by the customer |
| **Allergens** | Complies with Regulation (EU No.1169/2011) |
| **Suitability Data** | Kosher, FDA Certified |
| **GMO Statement** | The product and the raw material used to produce said product are free from GMO |
| **BSE / TSE Status** | BSE / TSE Free |
| **Irradiation Status** | Non-irradiated |
| **Remarks** | We hereby declare that our facility conforms to ISO/HACCP and that all necessary measures are in place to avoid cross contamination. |

ECOARTS
